# Proposal to classify *Ralstonia solanacearum* phylotype I strains as *Ralstonia nicotianae* sp. nov., and a genomic comparison between members of the genus *Ralstonia*

**DOI:** 10.3389/fmicb.2023.1135872

**Published:** 2023-03-22

**Authors:** Jun-Ying Liu, Jian-Feng Zhang, Han-Lian Wu, Zhen Chen, Shu-Ying Li, Hong-Mei Li, Cui-Ping Zhang, Yuan-Qing Zhou, Can-Hua Lu

**Affiliations:** ^1^College of Chemistry Biology and Environment, Yuxi Normal University, Yuxi, China; ^2^Institute of Biology and Environmental Engineering, Yuxi Normal University, Yuxi, China; ^3^College of Plant Health and Medicine, Qingdao Agricultural University, Qingdao, China; ^4^Yunnan Academy of Tobacco Agricultural Sciences, Kunming, China

**Keywords:** *Burkholderiaceae*, *Ralstonia*, sp. nov., phylotype I, phylogeny, *Ralstonia solanacearum* species complex, average nucleotide identity (ANI), digital DNA–DNA hybridization (dDDH)

## Abstract

A Gram-negative, aerobic, rod-shaped, motile bacterium with multi-flagella, strain RS^T^, was isolated from bacterial wilt of tobacco in Yuxi city of Yunnan province, China. The strain contains the major fatty acids of C_16:0_, summed feature 3 (C_16:1_*ω*7*c* and/or C_16:1_*ω*6*c*), and summed feature 8 (C_18:1_*ω*7*c* and/or C_18:1_*ω*6*c*). The polar lipid profile of strain RS^T^ consists of diphosphatidylglycerol, phosphatidylglycerol, phosphatidylethanolamine, and unidentified aminophospholipid. Strain RS^T^ contains ubiquinones Q-7 and Q-8. 16S rRNA gene sequence (1,407 bp) analysis showed that strain RS^T^ is closely related to members of the genus *Ralstonia* and shares the highest sequence identities with *R. pseudosolanacearum* LMG 9673^T^ (99.50%), *R. syzygii* subsp. *indonesiensis* LMG 27703^T^ (99.50%), *R. solanacearum* LMG 2299^T^ (99.28%), and *R. syzygii* subsp. *celebesensis* LMG 27706^T^ (99.21%). The 16S rRNA gene sequence identities between strain RS^T^ and other members of the genus *Ralstonia* were below 98.00%. Genome sequencing yielded a genome size of 5.61 Mbp and a G + C content of 67.1 mol%. The genomic comparison showed average nucleotide identity (ANIb) values between strain RS^T^ and *R. pseudosolanacearum* LMG 9673^T^, *R. solanacearum* LMG 2299^T^, and *R. syzygii* subsp. *indonesiensis* UQRS 627^T^ of 95.23, 89.43, and 91.41%, respectively, and the corresponding digital DNA–DNA hybridization (dDDH) values (yielded by formula 2) were 66.20, 44.80, and 47.50%, respectively. In addition, strains belonging to *R. solanacearum* phylotype I shared both ANIb and dDDH with strain RS^T^ above the species cut-off values of 96 and 70%, respectively. The ANIb and dDDH values between the genome sequences from 12 strains of *R. solanacearum* phylotype III (Current *R. pseudosolanacearum*) and those of strain RS^T^ were below the species cut-off values. Based on these data, we concluded that strains of phylotype I, including RS^T^, represent a novel species of the genus *Ralstonia*, for which the name *Ralstonia nicotianae* sp. nov. is proposed. The type strain of *Ralstonia nicotianae* sp. nov. is RS^T^ (=GDMCC 1.3533^T^ = JCM 35814^T^).

## 1. Introduction

The genus *Ralstonia*, a member of the family *Burkholderiaceae*, was named to accommodate *Alcaligenes eutrophus*, *Burkholderia pickettii*, and *B. solanacearum* (Ralston et al., 1973; Yabuuchi et al., 1995). Later, several novel species were described, and further studies suggested that the *Ralstonia* strains could be separated into two distinct groups (De Baere et al., 2001). Therefore, the genus *Wautersia* was created to accommodate the *R. eutropha* lineage (Vaneechoutte et al., 2004), which has been transferred to the genus *Cupriavidus* (Vandamme and Coenye, 2004). According to the List of Prokaryotic names with Standing in Nomenclature^1^ (Parte et al., 2020), the genus *Ralstonia* currently comprises seven species with validly published names, including *R. mannitolilytica*, *R. insidiosa*, *R. pickettii*, *R. pseudosolanacearum*, *R. solanacearum*, *R. syzygii*, and *R. wenshanensis* (Safni et al., 2014; Meier-Kolthoff et al., 2022; Lu et al., 2022a). Recently, we proposed three novel species of the genus *Ralstonia*, including *R. chuxiongensis*, *R. mojiangensis*, and *R. soli* (Lu et al., 2023).

Bacteria from the genus *Ralstonia* have a broad range of niches, including soil, water, plant tissues, and human specimens. The type strains of *R. chuxiongensis*, *R. mojiangensis*, *R. soli*, and *R. wenshanensis* have been isolated from tobacco planting soils (Lu et al., 2022a, 2023). The type strains of *R. mannitolilytica*, *R. insidiosa*, and *R. pickettii* were described as pathogens in various human infections (Ralston et al., 1973; De Baere et al., 2001; Coenye et al., 2003). *R. pseudosolanacearum*, *R. solanacearum*, and *R. syzygii* are critical economic pathogens of various plants (Yabuuchi et al., 1995; De Baere et al., 2001; Coenye et al., 2003; Remenant et al., 2010, 2011; Safni et al., 2014; Zulperi et al., 2014; Lu et al., 2021).

The pathogen responsible for plant bacterial wilt was first named *Bacillus solanacearum* (Smith, 1896) and then successively placed into the genera *Bacterium*, *Phytomonas*, *Xanthomonas*, *Pseudomonas*, and *Burkholderia*, before being transferred into the current genus, *Ralstonia* (Yabuuchi et al., 1995; Paudel et al., 2020). Although classified as *R. solanacearum*, distinct strains from diverse plants from various regions showed DNA–DNA hybridization values below the species cut-off value of 70%, thus *R. solanacearum* is considered a species complex (Genin and Denny, 2012). Based on genetic analysis of the 16S-23S internal transcribed spacer (ITS) region, endoglucanase (*egl*), and *hrpB* gene sequences, the *R. solanacearum* species complex was divided into four phylogenetically distinct groups, named phylotypes, which roughly reflect the strains’ geographical origins: phylotype I strains are often sampled from Asia; phylotype II strains are isolated from the Americas, which are further subdivided into three subgroups (IIA, IIB, and IIC) (Poussier et al., 2000; Sharma et al., 2022); phylotype III are obtained from Africa; and phylotype IV are collected from south-east Asia (Prior and Fegan, 2005; Villa et al., 2005). Furthermore, Safni et al. (2014) classified the phylotype II strains as the species *R. solanacearum*, phylotypes I and III strains as the species *R. pseudosolanacearum,* and the phylotype IV strains as *R. syzygii*. The phylotype IV strains were initially proposed as *Pseudomonas syzygii* by Yabuuchi et al. (1995), transferred into the genus *Ralstonia* in 2004, and further sub-classified into three subspecies, *R. syzygii* subsp. *syzygii*, *R. syzygii* subsp. *indonesiensis*, and *R. syzygii* subsp. *celebesensis* (Safni et al., 2014). Recently, based on 16S rRNA gene analysis and genome sequence comparisons, Fluit et al. (2021) found that several well-known strains of phylotype I, including GMI1000, OE1-1, EP1, and CQPS-1, might represent an unnamed species within the genus *Ralstonia*.

In the present study, a novel pathogenic bacterium, RS^T^, belonging to *Ralstonia* was isolated from tobacco bacterial wilt. It was identified by comparing its physiological and chemical characteristics, its 16S rRNA gene, and its whole genome with those of closely related type strains. Consequently, the name *Ralstonia nicotianae* sp. nov. is proposed for phylotype I strains and the type strain is RS^T^ (=GDMCC 1.3533^T^ = JCM 35814^T^).

## 2. Materials and methods

### 2.1. Sampling site and bacterial culture

**Figure 1:**
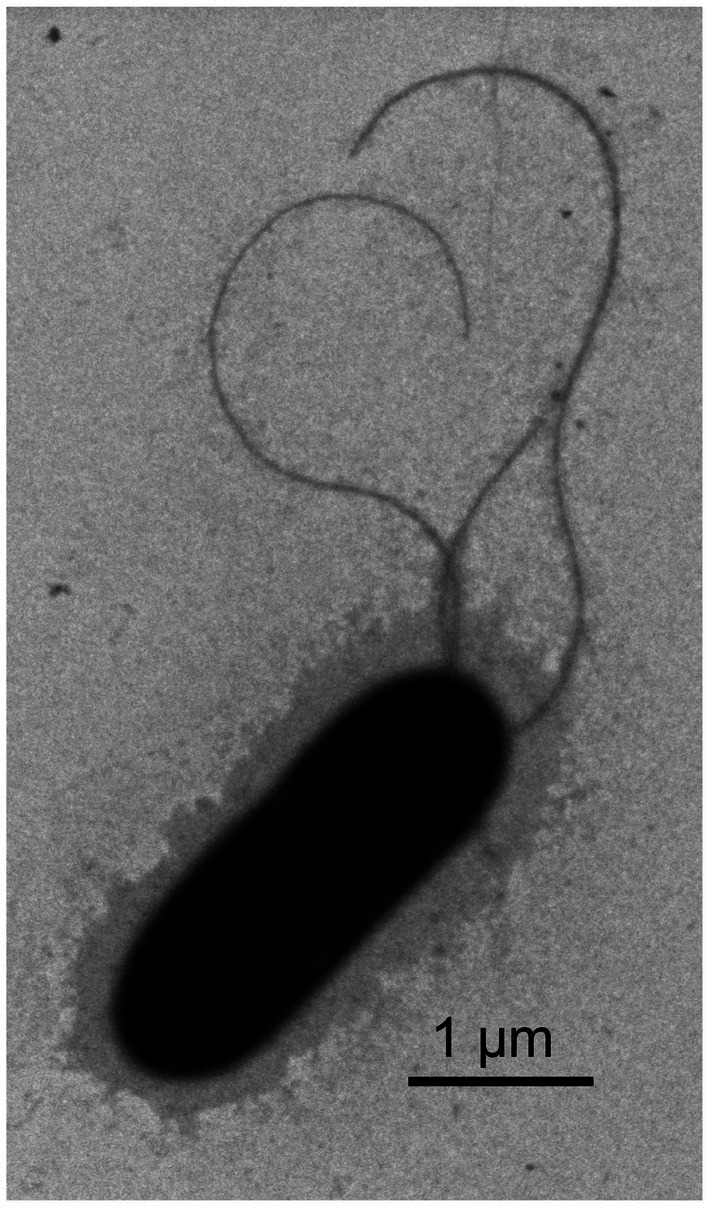
Transmission electron micrograph showing the morphology of strain RS^T^. The bar represents 1 μm.

Strain RS^T^ was isolated from bacterial wilt of flue-cured tobacco sampled in 2018 in Yuxi, Yunnan, China. The approximate geographical coordinates were 102°30′2.22″E, 24°14′22.5″N. The site is 1,630 m above sea level and has a central semi-humid subtropical cool winter plateau monsoon climate, with an average annual temperature of 15–16°C. We used the symptomatic leaves for pathogen isolation. First, the petiole part was surface-disinfected with 75% (v/v) ethanol for 5 min. Second, we removed tissues, including the epidermis layers, palisade, and spongy tissues, other than the leaf vein vascular bundle. Third, after removing the 2–3 cm part at the base, the remaining leaf vein vascular bundle was cut into several pieces and soaked in a sterile petri dish containing 10 ~ 20 mL of distilled water for 5–10 min. Finally, 100 μL of bacterial suspension with 5–6 times 10-fold dilution were spread onto a Tripheny tetrazolium chloride (TZC) agar plate. The dish was incubated under aerobic conditions at 28°C. After 36–48 h, typical circular colonies had formed, with broad white edges, strong fluidity, and a pink to light thin red liquid in the middle. The isolate was named RS^T^ and streaked onto TZC agar at 28°C and purified three times (Figure 1). Strain RS^T^ was stored in 20% (v/v) glycerol at −80°C for long-term preservation. It has been deposited at the Guangdong Microbial Culture Collection Center (GDMCC 1.3533^T^) and the Japan Collection of Microorganisms (JCM 35814^T^). The species type strains, including *R. solanacearum* LMG 2299^T^, *R. pseudosolanacearum* LMG 9673^T^, *R. syzygii* subsp. *indonesiensis* LMG 27703^T^, *R. syzygii* subsp. *syzygii* LMG 10661^T^, and *R. syzygii* subsp. *celebesensis* LMG 27706^T^, were collected from the BCCM/LMG Bacteria Collection (Table 1).

**Table 1:** Strain list used in this study.

Species/Subspecies	Strain	Source	Country	Bioproject No.	Assembly No.	16S rRNA	References
*Ralstonia nicotianae*	RS^T^(=GDMCC 1.3533^T^ = JCM 35814^T^)	Tobacco	China	PRJNA594457	GCA_018243235.1	ON797093.1	This study
	FQY_4	Tobacco	China	PRJNA182081	GCA_000348545.1	CP004012.1	Cao et al. (2013)
	CQPS-1	Tobacco	China	PRJNA331070	GCA_002220465.1	CP016914.1	Liu et al. (2017)
	GMI1000	Tomato	France	PRJNA13	GCA_000009125.1	AL646052.1	Salanoubat et al. (2002)
	EP1	Eggplant	China	PRJNA288736	GCA_001891105.1	CP015115.1	Li et al. (2016)
*R. pseudosolanacearum*	LMG 9673^T^(= NCPPB 10229^T^ = NCPPB 1029^T^ = UQRS 461^T^)	*Pelargonium capitatum*	Africa	PRJNA735438	GCA_024925465.1	CP103852.1	Safni et al. (2014) and Lu et al. (2023)
	RUN2474(=UW774)	Potato	Madagascar	PRJNA591018	GCA_014884685.1	CP051169.1	Steidl et al. (2021)
	RUN2279(=UW773)	Potato	Madagascar	PRJNA591018	GCA_014884705.1	CP051171.1	Steidl et al. (2021)
	CMR15(=CFBP6941)	Tomato	Cameroon	PRJEA50681	GCA_000427195.1	FP885895.1	Remenant et al. (2010)
	UW386	Soil	Nigeria	PRJNA531204	GCA_006088755.1	CP039339.1	Steidl et al. (2021)
*R. solanacearum*	LMG 2299^T^(=A. Kelman 60-1^T^ = ATCC 11696^T^ = CCUG 14272^T^ = CFBP 2047^T^ = CIP 104762^T^ = DSM 9544^T^ = ICMP 5712^T^ = JCM 10489^T^ = K60-1^T^ = NCAIM B.01459^T^ = NCPPB 325^T^ = NRRL B-3212^T^)	Tomato	USA	PRJNA381968	GCA_002251695.1	EF016361.1	Yabuuchi et al. (1995) and Remenant et al. (2012)
	CFBP2957_II-A	Tomato	French	PRJEA50685	GCA_000197855.1	FP885897.1	Remenant et al. (2010)
	Po82_II-B	Potato	Mexico	PRJNA66837	GCA_000215325.1	CP002819.1	Xu et al. (2011)
*R. syzygii*	LLRS-1	Tobacco	China	PRJNA594455	GCA_018243215.1	CP046729.1	Lu et al. (2021)
*R. syzygii* subsp*. celebesensis*	LMG 27706^T^(=DSM 27477^T^ = R-46908^T^ = UQRS 627^T^)	Banana	Indonesia	N/A	N/A	KC757073.1	Remenant et al. (2010) and Safni et al. (2014)
*R. syzygii* subsp*. celebesensis*	UGMSS_Db01	Banana	Indonesia	PRJNA580360	GCA_016743075.1	CP068285.1	Prakoso et al. (2022)
	R 229 (=ICMP 10001 = T 389 = LMG 27886)	Banana	Indonesia	PRJNA53877	N/A	N/A	Remenant et al. (2011) and Safni et al. (2014)
*R. syzygii* subsp*. indonesiensis*	LMG 27703^T^(=DSM 27478^T^ = PSI 7^T^ = R-46900^T^ = UQRS 464^T^)	Tomato	Indonesia	PRJEA50683	GCA_000283475.1	KC757057.1	Remenant et al. (2010) and Safni et al. (2014)
*R. syzygii* subsp*. syzygii*	LMG 10661^T^(=ATCC 49543^T^ = CCUG 32781^T^ = DSM 7385^T^ = ICMP 10915^T^ = NCPPB 3446^T^ = R 001^T^)	Clove	Indonesia	PRJEB43925	GCA_919592095.1	U28237.1	Remenant et al. (2011) and Safni et al. (2014)
*R. chuxiongensis*	21YRMH01-3^T^ (=GDMCC 1.3534^T^ = JCM)	Soil	China	PRJNA839150	GCA_024158925.1	ON844322.1	Lu et al. (2023)
*R. mojiangensis*	21MJYT02-10^T^ (=GDMCC 1.3531^T^ = JCM)	Soil	China	PRJNA839150	GCA_023955645.1	ON797091.1	Lu et al. (2023)
*R. soli*	21MJYT02-11^T^ (=GDMCC 1.3532^T^ = JCM)	Soil	China	PRJNA839150	GCA_023955655.1	ON797092.1	Lu et al. (2023)
*R. wenshanensis*	56D2^T^(=CCTCC AB 2021466^T^ = GDMCC 1.2886T = JCM 35178T 466^T^)	Soil	China	PRJNA735438	GCA_021173085.1	MZ399217.1	Lu et al. (2022a)
*R. pickettii*	K-288^T^(=JCM 5969^T^ = ATCC 27511^T^ = CCUG 3318^T^ = CFBP 2459^T^ = CIP 73.23^T^ = DSM 6297^T^ = HAMBI 2158^T^ = LMG 5942^T^ = NBRC 102503^T^ = NCTC 11149^T^)	Human	USA	PRJNA686860	GCA_016466415.2	LN681565.1	Yabuuchi et al. (1995) and Daligault et al. (2014)
*R. mannitolilytica*	LMG 6866^T^(=JCM 11284^T^ = CCUG 38408^T^ = CCUG 45027^T^ = DSM 17512^T^ = NCIB 10805^T^ = NCIMB 10805^T^)	Human blood	England	PRJEB43925	GCA_905397375.1	AJ270258.1	De Baere et al. (2001)
*R. insidiosa*	LMG 21421^T^(=CCUG 46789^T^ = DSM 17714^T^ = AU2944^T^)	Human sputum	USA	PRJNA563568	GCA_008801405.1	AF488779.1	Coenye et al. (2003)
*Cupriavidus necator*	N-1^T^(=ATCC 43291^T^ = CCUG 52238^T^ = CIP 103161^T^ = DSM 13513^T^ = LMG 8453^T^)	Soil	USA	PRJNA67893	GCA_000219215.1	NR_102851.1	Poehlein et al. (2011)

### 2.2. 16S rRNA sequencing, and phylogenetic and genomic comparisons

The genomic DNA of strain RS^T^ was extracted from a 2.0 mL bacterial culture using a bacterial DNA isolation kit (catalog number D3107; GBCBIO Technologies Inc., Guangzhou, China). The universal primer pair 27F and 1492R was used to amplify the 16S rRNA gene sequence (Clayton et al., 1995). The amplicon was ligated into the pMD19-T vector (Takara, Dalian, China) and sequenced following the Sanger method at Sangon Biotech Inc. (Shanghai, China). The sequences were assembled using Contig Express (Invitrogen, Waltham, MA, United States). The sequence similarity of the 16S rRNA gene from RS^T^ was calculated using the EzBiocloud database^2^ (Yoon et al., 2017). Closely related sequences of type species within the genus *Ralstonia* were collected from the GenBank database^3^ (Sayers et al., 2021) and aligned using MUSCLE mode in the Unipro UGENE version 33.0 (Unipro, Novosibirsk, Russia) (Table 1). Trimmed sequences were used for sequence identity calculations employing the Clustal Omega multiple alignment package in the EMBL-EBI database (Sievers and Higgins, 2021). Phylogenetic trees were constructed using the maximum-likelihood (ML), neighbor-joining (NJ), and minimum-evolutions (ME) algorithms, with bootstrap values of 1,000 replications in MEGA version 11 software (Kumar et al., 2016).

For genome sequencing, the glycerol suspension was streaked onto an agar plate containing Casamino Acid-peptone-glucose (CPG) medium (Kelman, 1954). A single colony was inoculated into CPG broth and grown for 24 h at 28°C at 180 r/min, followed by centrifugation at 10,000 × *g* for 10 min and washing with distilled water twice (Lu et al., 2021). The total DNA of strain RS^T^ was isolated using an EZNA bacterial DNA kit (Omega Bio-tek, Winooski, VT, United States). The genome of strain RS^T^ was sequenced using the PacBio RS II platform (Menlo Park, CA, United States) and the Illumina HiSeq 4,000 platform (San Diego, CA, United States) at the Beijing Genomics Institute (BGI, Shenzhen, China). Four SMRT cell Zero-Mode Waveguide arrays for sequencing were used by the PacBio platform to generate the subreads set. PacBio subreads of length < 1 kb were removed (Utturkar et al., 2014). The program Pbdagcon was used for self-correction (Chin et al., 2013). The draft genomic unitigs, which are uncontested groups of fragments, were assembled using the Celera Assembler against a high quality corrected circular consensus sequence subread set (Berlin et al., 2015). To improve the accuracy of the genome sequences, GATK (Poplin et al., 2018) and SOAP tool packages (SOAP2, SOAPsnp, SOAPindel) (Li et al., 2008, 2009) were used to make single-base corrections. Finally, to trace the presence of any plasmid, the filtered Illumina reads were mapped using SOAP to the bacterial plasmid database.^4^

Genetic comparisons between strain RS^T^ and type strains of the genus *Ralstonia* were tested based on average nucleotide identity (ANI) and digital DNA–DNA hybridization (dDDH) values using the webserver JSpecies WS version 3.9.6 (Richter et al., 2015) and the Genome-to-Genome Distance Calculator version 3.0 (Meier-Kolthoff et al., 2022), respectively. We conducted the phylogenomic analysis based on genome sequences of strain RS^T^ and thirteen closely related strains in the genus *Ralstonia* using the Type (Strain) Genome Server (Meier-Kolthoff et al., 2022). Strain *Cupriavidus necator* N-1^T^ served as an outgroup. To confirm the systematic classification of all *Ralstonia* strains in the GenBank database that were closely related to RS^T^, ANI and dDDH values were tested between genome sequences of strain RS^T^ and *R. pseudosolanacearum* LMG 9673^T^ and those from the strains mentioned above. For a quick calculation, ANI values were primarily tested using the FastANI calculator in the Genome Taxonomy Database (GTDB) (Jain et al., 2018; Parks et al., 2021). Strains with FastANI values between 93 and 97% were selected further to calculate BLAST-based ANI (ANIb), MUMmer-based ANI (ANIm), and dDDH values as described above.

For gene content analysis, the whole genome sequence of strain RS^T^ and those of closely related species in the genus *Ralstonia* were obtained from the GenBank database (Sayers et al., 2021), genes were predicted using GeneMarkS version 4.28 (Besemer et al., 2001) and the genome comparisons were performed using OrthoVenn2, with a threshold e-value of 1e-5 and inflation of 1.5 (Xu et al., 2019).

The type three effectors (T3Es) of strain RS^T^ were scanned using the Ralsto T3E database (Peeters et al., 2013; Sabbagh et al., 2019). To analyze the core effector repertoires of plant pathogenic *Ralstonia* species, we compared the T3Es in *Ralstonia* strains with the complete genome sequences from four phylotypes, including five strains of phylotype I, four strains of phylotype II, five strains of phylotype III, and three strains of phylotype IV. In addition, the secondary metabolites and antibiotic gene clusters were analyzed *in silico* using the online web server antiSMASH bacterial version 6.1.1 (Blin et al., 2021).

### 2.3. Phenotypic and chemotaxonomic analysis

According to the 16S rRNA gene analysis and genomic comparisons, we used eight *Ralstonia* strains, including RS^T^, GMI1000, CQPS-1, *R. pseudosolanacearum* LMG 9673^T^, *R. solanacearum* LMG 2299^T^, *R. syzygii* subsp*. celebesensis* LMG 27706^T^, *R. syzygii* subsp*. indonesiensis* LMG 27703^T^, and *R. syzygii* subsp*. syzygii* LMG 10661^T^, to compare the phenotypic properties and chemotaxonomic features.

The protocol used to analyze the strains’ physiological and chemical characteristics was as described previously (Lu et al., 2022a). Culture media, including CPG agar (Kelman, 1954), Kelman’s TZC (García et al., 2019), Luria-Bertani (LB) agar (LA), modified selective medium South Africa (mSMSA) agar (Elphinstone et al., 1996), nutrient agar (NA), potato dextrose agar (PDA), and trypticase soy agar (TSA, BD Difco, Franklin Lakes, NJ, United States), were used to analyze the growth of the strains when incubated at 28°C for 5 days. Cells grown on TSA at 28°C for 48 h were subjected to morphological observation using transmission electron microscopy (HT7700; HITACHI, Tokyo, Japan). The motilities of the tested strains were detected on modified semi-solid motility media (SMM) containing 0.35% agar (Kelman and Hruschka, 1973). A Gram-staining kit (D008-1-1; Nanjing Jiancheng Bioengineering Institute, Nanjing, China) was used stain the strains, which was detected under a light microscope (Axio imager.Z2; Zeiss, Oberkochen, Germany). The oxygen requirement assay was conducted by streaking the test strain on TSA slope covered with mineral oil and incubating at 28°C for 5 days (Lu et al., 2022b).

To analyze the chemical sensitivity, enzymatic activity, carbon source, and substrate utilization of the tested strains, the Biolog GEN III Microplate system, API 20NE, and API ZYM kits (bioMérieux, Marcy-l’Étoile, France), respectively, were used according to the manufacturer’s instructions. Oxidase activities of the strains were tested using 1% (w/v) tetramethyl-*p*-phenylenediamine, with *Escherichia coli* K-12 strain MG 1655 as the negative control and *Pseudomonas lijiangensis* LJ2^T^ as the positive control. The production of bubbles detected catalase activity after adding a drop of 3% (v/v) H_2_O_2_ (Lu et al., 2022a). Cells from the tested strains grown on TSA at 28°C for 48 h were harvested to analyze their fatty acid profile (Sasser, 1990). Polar lipids and isoprenoid quinones of three proposed type strains were extracted and analyzed as described previously (Minnikin and Abdolrahimzadeh, 1971; Collins and Jones, 1981).

## 3. Results and discussion

### 3.1. 16S rRNA phylogeny

**Figure 2:**
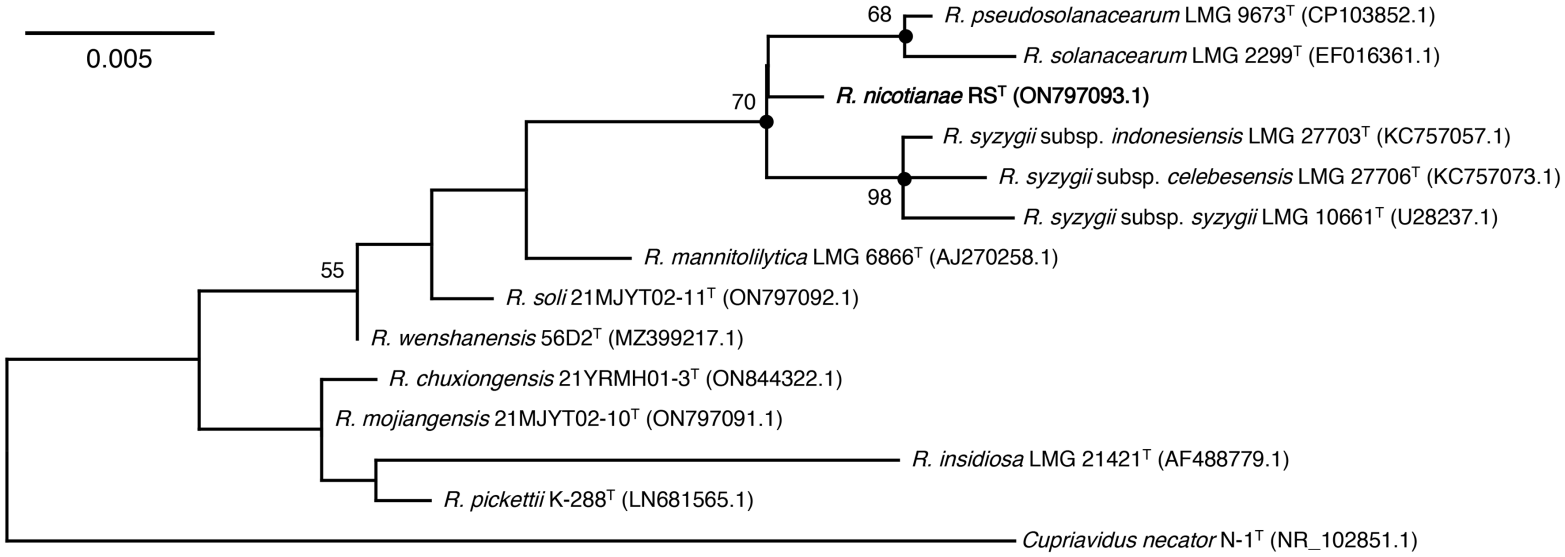
Maximum-likelihood phylogenetic tree based on 16S rRNA sequences of strain RS^T^ and type species in the genus *Ralstonia*. The tree was generated using MEGA 11.0. Bootstrap values (≥50) are shown at branching points as percentages of 1,000 replicates. ^T^ indicates the type strain. Closed circles indicate that the corresponding nodes were also recovered in the trees constructed with neighbor-joining and maximum-parsimony. Strain RS^T^ is indicated in bold. *Cupriavidus necator* N-1^T^ is used as an outgroup. The GenBank accession numbers are shown in parentheses. Bar, 0.005 substitutions per nucleotide position.

16S rRNA gene sequence analysis showed that strain RS^T^ was closely related to the type species in the genus *Ralstonia* and shared the highest 16S rRNA sequence identity (99.50%) with those of *R. pseudosolanacearum* LMG 9673^T^ and *R. syzygii* subsp. *indonesiensis* LMG 27703^T^, followed by *R. solanacearum* LMG 2299^T^ (99.28%) and *R. syzygii* subsp. *celebesensis* LMG 27706^T^ (99.21%). The 16S rRNA gene sequence identities between strain RS^T^ and the other type strains of the genus *Ralstonia* were all below 98.00% (Table 2). The ML phylogenetic tree based on 16S rRNA gene sequences revealed that strain RS^T^ formed a coherent cluster with type species of *R. pseudosolanacearum* LMG 9673^T^, *R. solanacearum* LMG 2299^T^, and three subspecies, including *R. syzygii* subsp. *indonesiensis* LMG 27703^T^, *R. syzygii* subsp. *celebesensis* LMG 27706^T^, and *R. syzygii* subsp. *syzygii* LMG 10661^T^ (Figure 2). The NJ and MP trees also indicated that strain RS^T^ was consistently separated from its closely related type species of *R. pseudosolanacearum*, *R. solanacearum*, and *R. syzygii* (Supplementary Figures 1, 2).

**Table 2:** Genetic comparisons between strain RS^T^ and *R. pseudosolanacearum* LMG 9673^T^, and type species in the genus *Ralstonia*.

Strain	RS^T^						*R. pseudosolanacearum* LMG 9673^T^						G + C content^b^
	16S rRNAs^a^	ANIb^b^	ANIm^b^	DDH1^c^	DDH2^c^	DDH3^c^	16S rRNA^a^	ANIb^b^	ANIm^b^	DDH1^c^	DDH2^c^	DDH3^c^	
RS^T^	100.00	100.00	100.00	100.00	100.00	100.00	99.50	95.30	96.15	73.40	66.20	74.50	67.10
*R. pseudosolanacearum* LMG 9673^T^	99.50	95.23	96.14	73.40	66.20	74.50	100.00	100.00	100.00	100.00	100.00	100.00	66.60
*R. syzygii* subsp. *syzygii* LMG 10661^T^	97.18	91.18	93.00	51.40	48.80	51.10	96.97	90.96	92.73	47.70	47.30	47.50	66.50
*R. syzygii* subsp. *indonesiensis* LMG 27703^T^	99.50	91.41	92.84	65.70	47.50	63.00	99.28	90.93	92.50	58.60	45.60	56.50	66.30
*R. solanacearum* K60-1^T^	99.28	89.69	91.64	58.60	42.20	55.40	99.57	89.62	91.56	57.60	41.80	54.50	66.40
*R. mannitolilytica* LMG 6866^T^	97.40	83.00	86.72	36.30	27.70	33.30	97.33	83.00	86.77	35.10	27.60	32.30	65.80
*R. soli* 21MJYT02-11^T^	97.62	83.08	86.87	33.00	27.60	30.70	97.48	83.20	86.87	31.60	27.70	29.60	64.10
*R. insidiosa* CCUG 46789^T^	97.26	82.80	86.46	34.00	26.90	31.30	97.33	82.82	86.49	32.30	26.90	30.00	63.70
*R. wenshanensis* 56D2^T^	97.69	82.49	86.36	35.40	26.70	32.30	97.55	82.56	86.38	33.20	26.70	30.60	63.70
*R. pickettii* K-288^T^	97.26	82.57	86.27	36.60	26.60	33.10	97.11	82.69	86.26	35.20	26.50	32.10	63.90
*R. chuxiongensis* 21YRMH01-3^T^	97.26	82.26	86.22	34.70	26.30	31.70	97.11	82.37	86.22	32.80	26.40	30.20	63.50
*R. mojiangensis* 21MJYT02-10^T^	97.40	82.36	86.24	35.20	26.20	32.00	97.26	82.36	86.28	33.10	26.30	30.40	63.60

Data are presented as a percentage.

a The 16S rRNA sequence similarities were calculated using the Clustal Omega multiple alignment package in the EMBL-EBI database (Sievers and Higgins, 2021).

b The ANI (ANIb and ANIm) and overall G + C content values were generated using the online service JSpeciesWS (Richter et al., 2015).

c The dDDH values were yielded using the Genome-to-Genome Distance Calculator (Meier-Kolthoff et al., 2022). 16S rRNAs = 16S rRNA sequence similarity. DDH1 = formula 1. DDH2 = formula 2. DDH3 = formula 3.

### 3.2. Genome features

**Figure 3:**
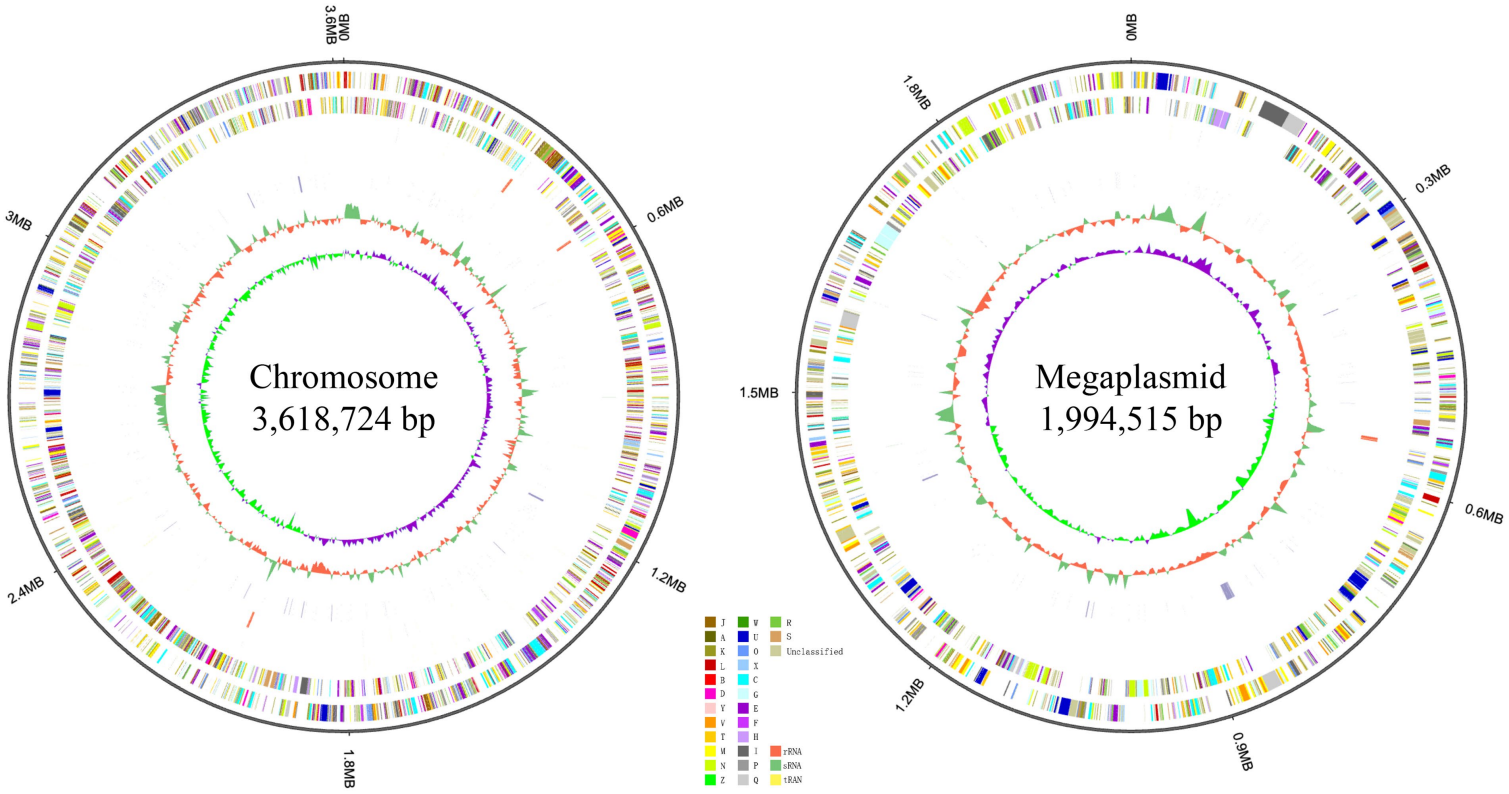
Localization of the principal genomic elements predicted in strain RS^T^. For each figure, from outer to inner: ring 1–genome size; ring 2–forward strand gene, colored according to the cluster of orthologous groups (COG) classification; ring 3–reverse strand gene, colored according to the cluster of orthologous groups (COG) classification; ring 4–forward strand ncRNA; ring 5–reverse strand ncRNA; ring 6–repeat elements; ring 7–G + C content; ring 8–GC-SKEW. Chromosome and megaplasmid of strain RS^T^ are presented on the left and right, respectively.

Genome sequencing yielded 3,971,364 reads and 492 Mb of clean data, with a genome coverage depth of 87×. The overall G + C content of strain RS^T^ was 67.10 mol%, which is in the range of 63.0–67.3 mol% (avg. 65.86 mol%) for *Ralstonia* species (Lu et al., 2022a). The assembled genome of strain RS^T^ comprised 5,613,239 bp in two replicons: a circular chromosome of 3,618,724 bp and a megaplasmid of 1,994,515 bp. Strain RS^T^ contained 5,105 predicted genes; 58 transfer RNA (tRNA) genes; four sets of 5S rRNA, 16S rRNA, and 23S rRNA genes; and 23 small RNA (sRNA) genes (Figure 3).

Genome comparisons between strain RS^T^ and type strains of the genus *Ralstonia* showed that *R. pseudosolanacearum* LMG 9673^T^ had the highest ANIb of 95.23% and ANIm of 96.14% in comparison with strain RS^T^, which was located in the species’ border cut-off value of 95–96%, followed by *R. syzygii* subsp. *syzygii* LMG 10661^T^ (ANIb = 91.18% and ANIm = 93.00%), *R. syzygii* subsp. *indonesiensis* LMG 27703^T^ (ANIb = 91.41% and ANIm = 92.84%), and *R. solanacearum* K60-1^T^ (ANIb = 89.69% and ANIm = 91.64%) (Table 2). The ANI values between the seven other type strains of the genus *Ralstonia* and strain RS^T^ were below 90.00%. The dDDH values yielded by formula two were used for analysis (Meier-Kolthoff et al., 2022). Data revealed that *R. pseudosolanacearum* LMG 9673^T^ shared the highest dDDH value with strains RS^T^ at 66.20%, which was below the 70% threshold value recommended for species description. The other 10 type strains had dDDH values with strain RS^T^ below 66.00% (Table 2).

**Figure 4:**
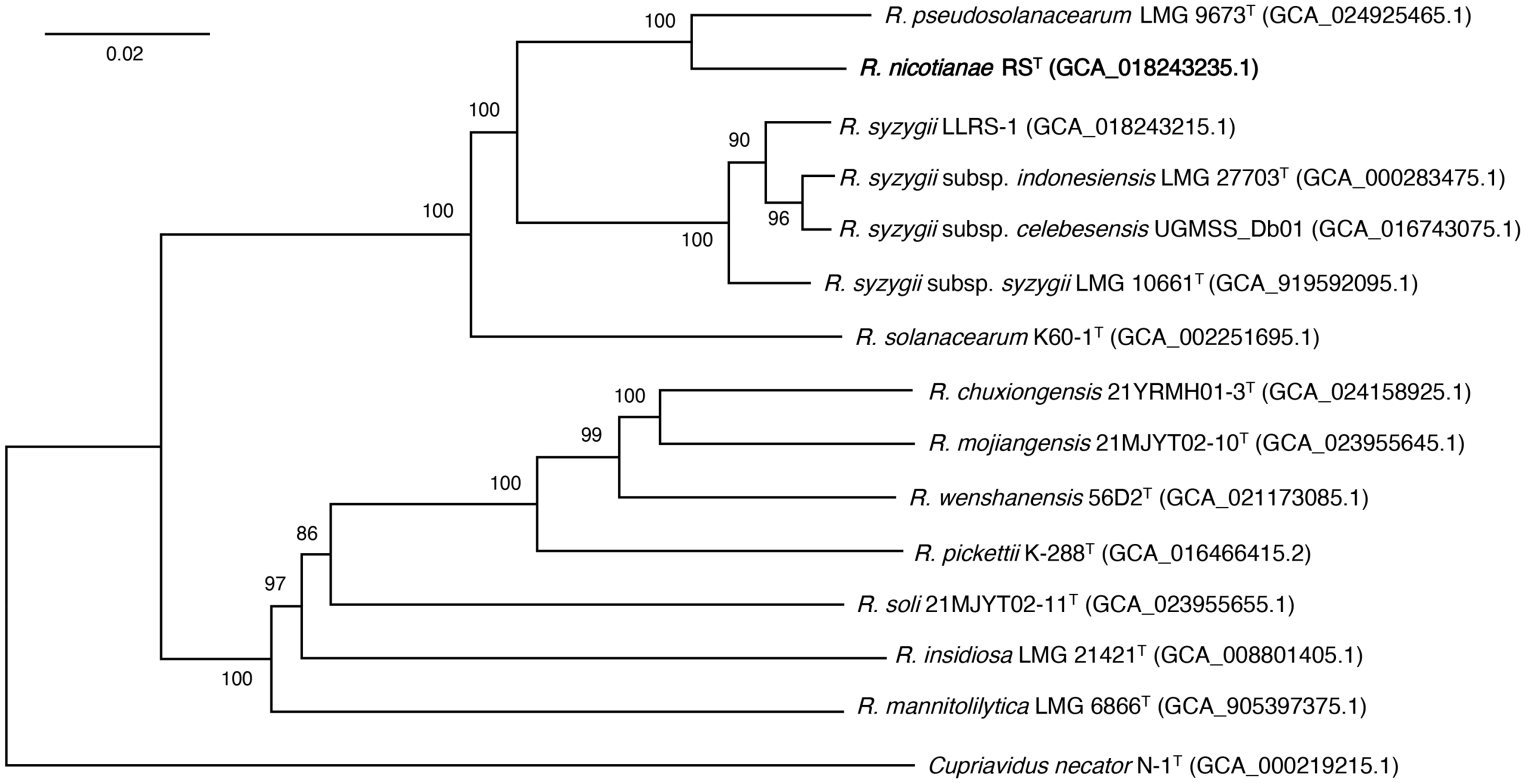
The phylogenomic tree showing the relationship between strain RS^T^ and its closely related strains in the genus *Ralstonia*. The phylogenomic tree was generated using the Type (Strain) Genome Server (Meier-Kolthoff and Göker, 2019). The numbers above branches are Genome BLAST Distance Phylogeny approach (GBDP) pseudo-bootstrap support values >50% from 100 replicates. ^T^ indicates the type strain. Strain RS^T^ is shown in bold. *Cupriavidus necator* N-1^T^ is used as an outgroup. Genomic assembly numbers are shown in parentheses. Bar, 0.02 changes per nucleotide position.

The phylogenomic analysis showed that 14 *Ralstonia* strains were divided into two main phylogenomic branches. Strain RS^T^ was positioned in the branch that contained three species, including *R. pseudosolanacearum*, *R. syzygii*, and *R. solanacearum*, which are the main causal agents of bacterial wilts in a wide range of plants. Strain RS^T^ formed a coherent cluster with type species of *R. pseudosolanacearum* LMG 9673^T^ at a supported bootstrap value of 100% (Figure 4). The results allowed us to conclude that strain RS^T^ represents a novel species in the genus *Ralstonia*. Interestingly, compared with strain *R. solanacearum* K60-1^T^, *R. syzygii* strains were genetically closer to strain RS^T^ and *R. pseudosolanacearum* LMG 9673^T^.

To confirm their systematic classifications, all *Ralstonia* strains closely related to RS^T^ whose genome sequences were available in the GenBank database were subjected to genome comparisons using the FastANI calculator in the Genome Taxonomy Database (GTDB) (Jain et al., 2018; Parks et al., 2021). Data showed that strain RS^T^ had FastANI values >95.00% with 211 *Ralstonia* strains. Among them, two hundred and one genomes shared FastANI values >97.00% and dDDH values ≥88.70% with RS^T^. In addition, they had FastANI values of 94.44–96.11% and dDDH values of 63.90–66.90% with *R. pseudosolanacearum* LMG 9673^T^ (Supplementary Table 1). These data suggested that these strains (phylotype I) are closely related to strain RS^T^. The systematic classifications of 10 strains of phylotype III, including CFBP3059, CMR15, NCPPB 216, UW386, RUN2279, RUN2587_UW776, RUN2474, NCPPB332_UW654, DGBBC1138_UW685, and CIP296_UW472, showed that they shared FastANI values below 97% with strain RS^T^, which was further confirmed using ANI (ANIb and ANIm) and dDDH tests. The results revealed that all strains had ANIb values ≤95.33% and dDDH values ≤67.10% with strain RS^T^ and shared ANIb values ≥95.65% and dDDH values ≥70.50% with *R. pseudosolanacearum* LMG 9673^T^ (Supplementary Tables 1, 2), supporting the view that these strains belong to the species of *R. pseudosolanacearum*. Based on the genome sequences of strains RS^T^, *R. pseudosolanacearum* LMG 9673^T^, and their closely related strains using the Type (Strain) Genome Server (Meier-Kolthoff et al., 2022), a phylogenomic tree also supported the view that strain RS^T^ and its closely associated strains of phylotype I formed a coherent cluster with five complete genome-sequenced *R. pseudosolanacearum* strains (Supplementary Figure 3). These results suggested that strain RS^T^ isolated from bacterial wilt of tobacco and its closely related strains of phylotype I represent a novel species in the genus *Ralstonia*.

### 3.3. Pan-genome profile and accessory genes

**Figure 5:**
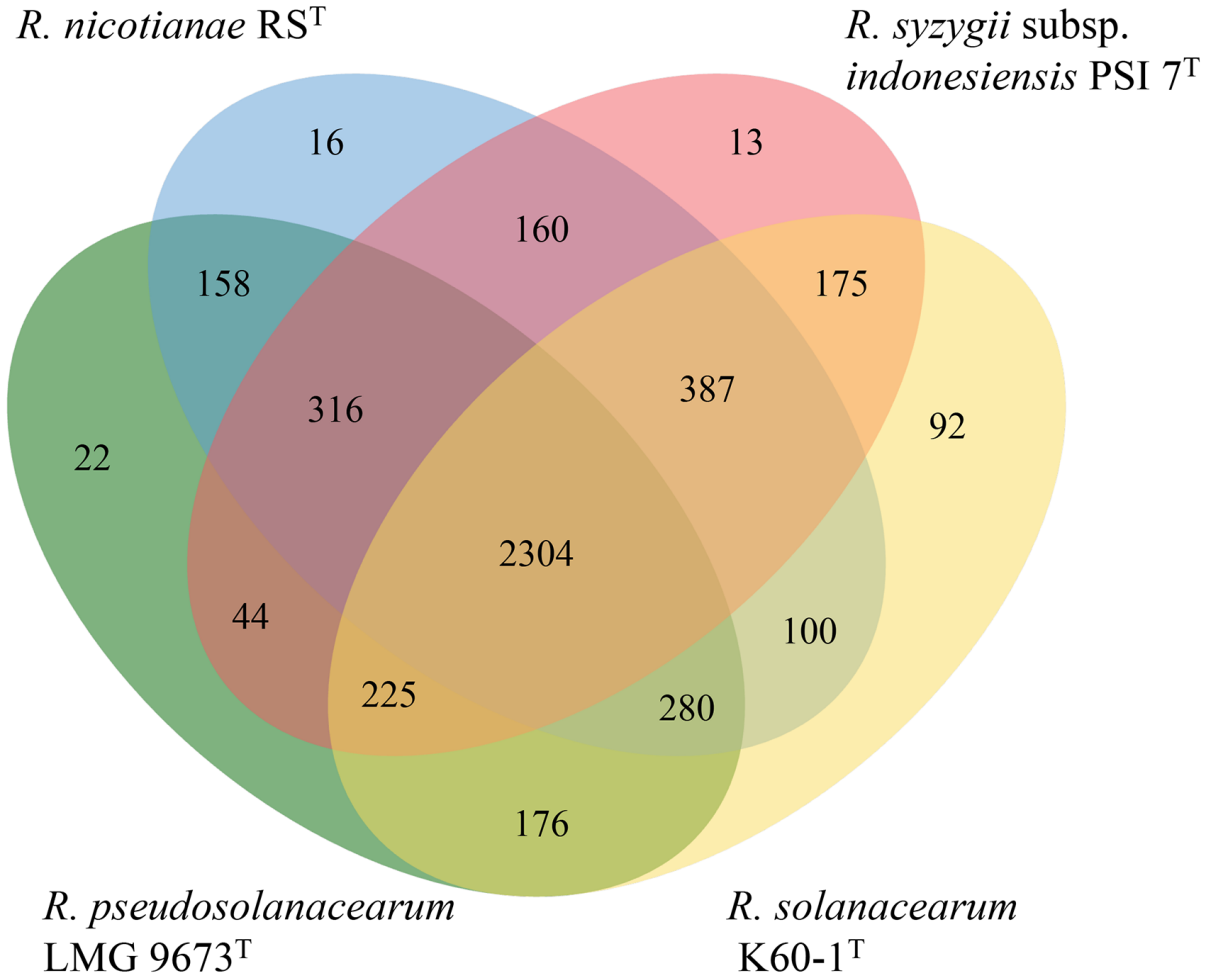
Venn diagram of the distribution of shared gene families (orthologous clusters) among four plant pathogenic strains of the *Ralstonia* (Xu et al., 2019).

Data revealed that 11 whole genome sequences from strains RS^T^ and type species in the genus *Ralstonia* had a total of 55,346 protein-coding genes, with an average of 5031.45 genes for each species. The protein coding sequences were grouped into 7,159 clusters, containing 4,564 orthologous clusters and 2,595 single-copy gene clusters. All strains shared 2,657 core genome orthologs, which are mainly involved in translation, cell shape regulation, phenylacetate catabolic process, and DNA binding transcription factor activity. Strains from seven non-plant pathogenic type species within the genus *Ralstonia* shared 252 specific core orthologous clusters, and strains from four plant pathogenic bacteria, including RS^T^, *R. pseudosolanacearum* LMG 9673^T^, *R. solanacearum* K60-1^T^, and *R. syzygii* PSI 7^T^, had 301 unique core orthologous gene clusters, which were absent in non-pathogenic *Ralstonia*. When we compared the four plant pathogenic bacteria, type strains formed 4,468 clusters, which included 2,304 core orthologs. In addition, strain RS^T^ had 16 specific protein family clusters consisting of 47 proteins, which were not present in type strains of the other three plant pathogenic *Ralstonia*. Eight unique clusters in strain RS^T^ were assigned to biological processes such as nucleobase-containing compounds, cellular aromatic and nitrogen compounds, macromolecules, cellular, primary, and heterocycle metabolic processes, and DNA transposition. Strain RS^T^ shared 158, 160, and 100 orthologous clusters with its closely related type strains *R. pseudosolanacearum* LMG 9673^T^, *R. syzygii* subsp. *indonesiensis* PSI 7^T^*, and R. solanacearum* K60-1^T^, respectively, suggesting that strain RS^T^ is more closely related to *R. pseudosolanacearum* LMG 9673^T^ and *R. syzygii* subsp. *indonesiensis* PSI 7^T^ than to *R. solanacearum* K60-1^T^ (Figure 5).

### 3.4. Core repertoire of T3Es

A total of 57 candidate T3Es, three pseudogenes (RipB, RipS5, and RipAT), and seven hypothetical effectors without assignment were found in strain RS^T^, among which 37 core T3Es were also found in five other closely related strains (Supplementary Table 3). A unique effector, RipAK, was present in strains of phylotype I, but absent in those belonging to phylotypes II, III, and IV. RipAK acts as a plant hypersensitive response suppressor by interacting with host pyruvate decarboxylases (PDCs), inhibiting their oligomerization and enzymatic activities (Sun et al., 2017; Wang et al., 2021). Two T3E candidates, RipBI and Hyp9, were conserved and unique among strains of phylotype II. The functional role of RipBI in *Ralstonia* is undefined; however, its homologous core effector XopX in *Xanthomonas* is a virulence factor and can both activate and suppress host immune responses in rice (Metz et al., 2005; Stork et al., 2015). Further study found that XopX induces immune responses by interacting with XopQ (Deb et al., 2020). RipBF is a phylotype IV-specific effector, which was only found in the tested *R. syzygii* strains; however, its functional roles remain to be determined. RipAP was absent from all *R. syzygii* strains, but was present in all tested strains of phylotypes I, II, and III. Although 35 candidate T3Es were present in all tested *R. pseudosolanacearum* strains, no specific effector was found among phylotype III strains, including LMG 9673^T^, CMR15, RUN2279, RUN2474, and UW386.

**Figure 6:**
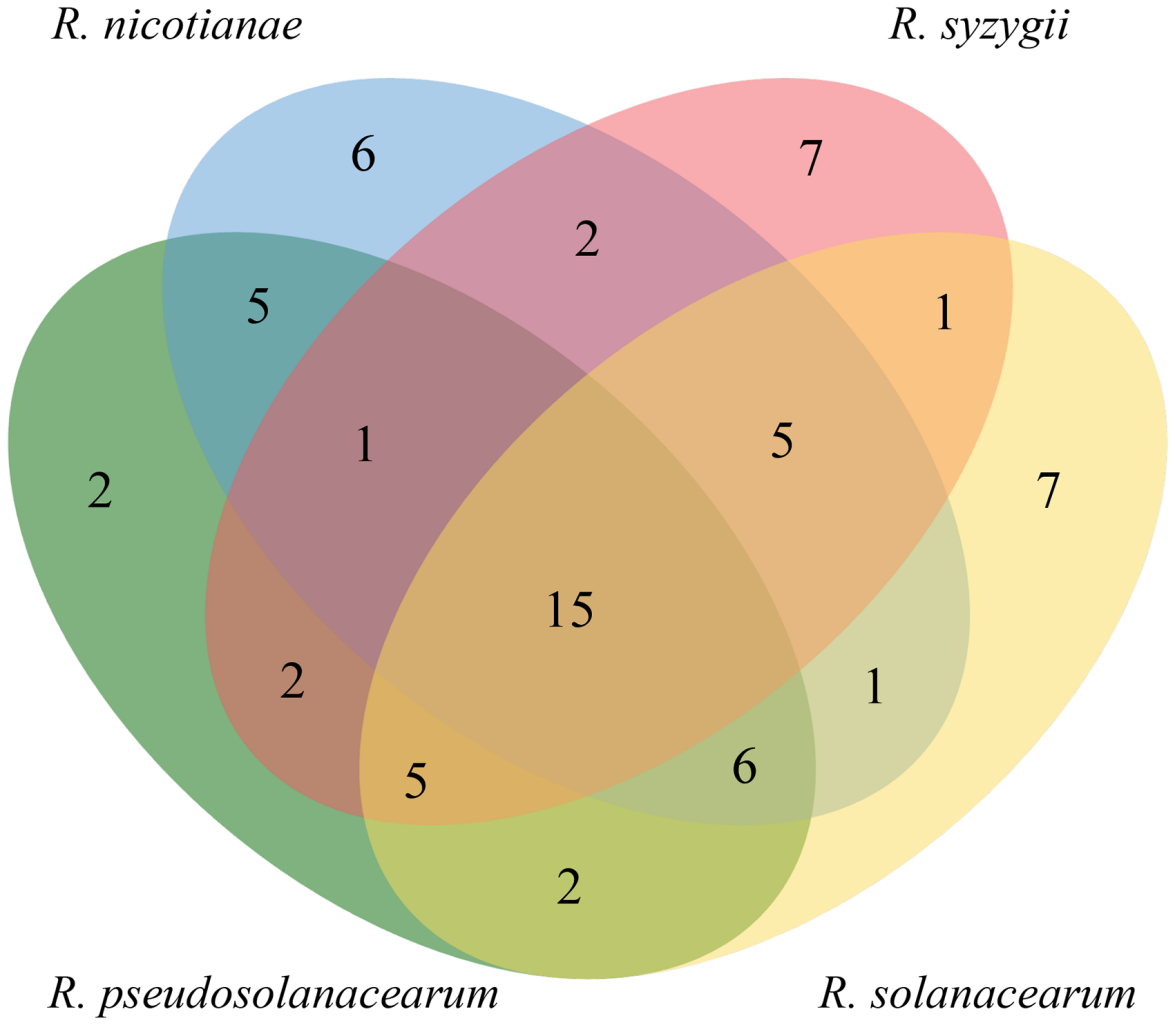
Venn diagram of the distribution of shared candidate T3Es (core effectors) among strains of four phylotypes (https://bioinfogp.cnb.csic.es/tools/venny/index.html).

In a Venn diagram analysis, we also found that 15 core T3Es, including RipAE, RipAJ, RipC1, RipAI, RipAC, RipB, RipAB, RipX, RipZ, RipU, RipAO, RipR, RipAQ, RipAM, and RipAN, were conserved among 18 tested *Ralstonia* strains (Figure 6). This suggested that these effectors play essential roles in pathogenicity. Indeed, Cong et al. (2022) have heterologously expressed the five core effectors of phylotype I strain P380 in the polymutant *Pseudomonas syringae* DC3000D36E. Their data showed that RipAE, RipC1, RipU, and RipW inhibited the reactive oxygen species (ROS) burst, while RipAQ did not. The core effectors, including RipAE, RipU, and RipW, induced cell death as well as upregulating mitogen-activated protein kinase (MAPK) cascades in *Nicotiana benthamiana* (Cong et al., 2022). Furthermore, RipAC suppresses the hypersensitive response triggered by avirulence effector RipAA of phylotype I strain GMI1000 (Nakano et al., 2020). Moreover, effector RipAC suppresses Nod-like receptor (NLR)-triggered immune responses by inhibiting the interaction between MAPKs and SGT1 (suppressor of G2 allele of SKP1 homolog) targets, and the phosphorylation of a MAPK target motif in the C-terminal domain of SGT1 (Nakano et al., 2020). Recently, Yu et al. (2022) found that RipAC inhibits pattern-triggered immunity by targeting E3 ubiquitin ligase plant U-box 4 (PUB4). Furthermore, core effector RipB acts as a major avirulence factor in *Nicotiana* species and suppresses the basal defense in susceptible hosts to promote pathogen infection (Nakano and Mukaihara, 2019; Cao et al., 2022). In addition, the nuclear-localized effectors RipAB and RipX are involved in plant immune response (Zheng et al., 2019; Sun et al., 2020). However, the functions of the other core effectors, including RipAJ, RipC1, RipAI, RipZ, RipAO, RipR, RipAM, and RipAN, still need to be determined.

### 3.5. Gene prediction involved in secondary metabolites

*In silico* secondary metabolite analysis predicted 12 gene clusters in the genome of strain RS^T^, referring to seven cluster types, including aryl polyene, furan, homoserine lactone (hserlactone), non-ribosomal peptide synthetase (NRPS), NRPS-like, ribosomally synthesized and post-translationally modified peptide product-like (RiPP-like), terpene, and type I polyketide synthase (T1PKS) (Supplementary Table 4). Among them, the taxonomically widespread aryl polyene, RiPP-like, and terpene biosynthetic gene clusters were also present in all tested *Ralstonia* strains (Nishida et al., 2005; Arnison et al., 2013; Cimermancic et al., 2014). Interestingly, more known secondary metabolite gene clusters were detected in the plant pathogenic *Ralstonia* species (10.87 clusters/strain) than in the non-pathogenic group (6.28 clusters/strain), which might suggest that more secondary metabolites remain to be uncovered in the latter group. Data revealed that the hserlactone and furan types were found in pathogenic *Ralstonia* strains. Both metabolite clusters shared less than 20% similarity to known gene clusters, suggesting that these gene clusters might encode novel metabolites and play significant roles in pathogenicity. The homoserine lactone gene cluster, which is usually involved in signal molecule production, participates in cell-to-cell communication processes. A recent study showed that the novel RasI/R quorum sensing system regulates phylotype I strain EP1 virulence. When the signal molecule N-(3-hydroxydodecanoyl)-homoserine lactone synthetic gene *rasI* was knocked out, the mutant lost virulence toward eggplants (Yan et al., 2022). The metabolites of NRPS, NRPS-like, and T1PKS types were also only found in plant pathogenic *Ralstonia*, except for *R. pickettii* K-288^T^, which only contained a T1PKS gene cluster. *In silico* metabolite analysis found that the beta-lactone-containing protease inhibitor (betalactone) and redox-cofactor types appeared to be conserved in non-pathogenic *Ralstonia* strains.

Siderophores are essential factors for both plant pathogens and beneficial microorganisms (Gu et al., 2020). Based on their chemical nature, siderophores are mainly classified into hydroxamate, catecholate, and carboxylate (Khan et al., 2018). Data revealed that strain RS^T^ carried three putative siderophore biosynthetic clusters. Firstly, the chromosome of strain RS^T^ encodes a micacocidin biosynthetic cluster (Kreutzer et al., 2011), which is conserved among phylotypes I, III, and IV strains, but absent in strains of phylotype II. However, a gene cluster accounting for siderophore yersiniabactin biosynthesis was only present in strains of phylotype II. Secondly, we detected a gene cluster that shared 18% similarity to the biosynthesis gene cluster of carboxylate siderophore staphyloferrin B on the megaplasmid of strain RS^T^ (Bhatt and Denny, 2004). Thirdly, a specific NRPS and/or NRPS-like gene cluster was conserved among tested five strains of phylotype I, with a size range of 48.51–51.11 kb. It consisted of two core biosynthetic genes, seven additional biosynthetic genes, two transport-related genes, six regulatory genes, and 16 other genes on the megaplasmid of strain RS^T^. It shared no sequence similarity with known gene clusters; however, the MIBiG comparison showed that it had the highest similarity score of 0.65 with the catecholate siderophore Amphi-enterobactin 1 biosynthetic gene cluster in the marine organism *Vibrio harveyi* BAA-1116 (McRose et al., 2018). However, the functional roles of this secondary metabolite biosynthetic cluster are uncharacterized.

In addition, nine out of 10 tested strains from phylotypes I and III shared a specific ralsolamycin biosynthetic cluster (100% similarity), consistent with a previous report that *R. solanacearum* K60-1^T^ does not encode this specific gene cluster (Spraker et al., 2016). The diffusible lipopeptide ralsolamycin was found in a survey of secondary metabolites that induced chlamydospore formation of fungi when co-cultured with strain GMI1000 (Spraker et al., 2016). The biosynthesis locus of ralsolamycin comprises a non-ribosomal peptide synthetase and polyketide synthase hybrid gene (*RmyA*) and an NRPS gene (*RmyB*) (Spraker et al., 2016). Data showed that the PhcBSR quorum sensing (QS) system regulates ralsolamycin production (Li et al., 2017, 2022). A recent study found that ralsolamycin induces the production of the fungal secondary metabolite bikaverin when *Fusarium fujikuroi* was exposed to ralsolamycin (Spraker et al., 2018), suggesting that this secondary metabolite can act as a communication chemical between bacteria and fungi.

### 3.6. Physiology and chemotaxonomy

In the API assays, strains of phylotype I, including RS^T^, GMI1000, and CQPS-1, shared more similarity to *R. pseudosolanacearum* LMG 9673^T^ and *R. solanacearum* LMG 2299^T^ for many phenotypic characteristics, e.g., all strains were positive for the reduction of potassium nitrate; hydrolysis of eculin; assimilation of D-glucose, potassium gluconate, malic acid, and trisodium citrate; and catalysis activity; and negative for indole production; the assimilation of arabinose, mannose, maltose, and adipic acid; and the activities of arginine dihydrolase, urease, protease, α-galactosidase, β-galactosidase, esterase lipase, valine arylamidase, cystine arylamidase, trypsin, α-chymotrypsin, β-glucuronidase, α-glucosidase, β-glucosidase, N-acetyl-β-glucosaminidase, α-mannosidase, and α-fucosidase. However, strains of phylotype I had unique phenotypic features. For example, *R. pseudosolanacearum* LMG 9673^T^ and *R. solanacearum* LMG 2299^T^ were negative for leucine arylamidase and acid phosphatase activity individually, whereas phylotype I strains were positive. In addition, *R. pseudosolanacearum* LMG 9673^T^ and *R. solanacearum* LMG 2299^T^ were positive for lipase and Naphthol-AS-BI-phosphohydrolase activity, respectively, and both of them were positive for glucose fermentation; however, all tested phylotype I strains were negative.

In the Biolog GEN III assays, tested phylotype I strains were more similar to *R. pseudosolanacearum* LMG 9673^T^ and *R. solanacearum* LMG 2299^T^ for several phenotypic characteristics. All strains were positive for the utilization of D-fructose-6-PO_4_, D-galacturonic acid, L-galactonic acid lactone, D-glucuronic acid and glucuronamide; were able to grow under conditions supplemented with rifamycin SV, lincomycin, vancomycin, tetrazolium violet, tetrazolium blue and potassium tellurite; were negative for the utilization of carbon sources such as D-maltose, D-trehalose, D-cellobiose, gentiobiose, sucrose, D-turanose, stachyose, D-raffinose, α-D-lactose, D-melibiose, β-methyl-D-glucoside, D-salicin, N-acetyl-D-glucosamine, N-acetyl-β-D-mannosamine, N-acetyl-D-galactosamine, N-acetyl neuraminic acid, L-rhamnose, inosine, D-sorbitol, D-mannitol, D-arabitol, myo-inositol, glycerol, D-serine, Gelatin, glycyl-L-proline, L-alanine, L-arginine, L-pyroglutamic acid, L-serine, D-gluconic acid, mucic acid, quinic acid, D-saccharic acid, p-hydroxy-phenylacetic acid, methyl pyruvate, D-lactic acid methyl ester, L-lactic acid, D-malic acid, bromo-succinic acid, α-hydroxy-butyric acid, α-keto-butyric acid, propionic acid, formic acid; and were unable to grow under conditions supplemented with inhibitory chemicals, including pH 5, 1% NaCl, 4% NaCl, 8% NaCl, 1% sodium lactate, fusidic acid, D-serine, troleandomycin, minocycline, guanidine HCl, niaproof 4, lithium chloride, sodium butyrate, sodium bromate. Furthermore, the data also showed that the tested phylotype I strains, including RS^T^, GMI1000, and CQPS-1, were more similar to *R. pseudosolanacearum* LMG 9673^T^. For example, these four strains were unable to use D-aspartic acid, L-histidine, α-keto-glutaric acid, γ-amino-butyric acid, β-hydroxy-D,L-butyric acid, and acetoacetic acid, whereas strain *R. solanacearum* LMG 2299^T^ was able to use these carbon sources. However, *R. pseudosolanacearum* LMG 9673^T^ tested positive for the use of two carbon sources, D-Mannose and L-Fucose and one inhibitory chemical (nalidixic acid), whereas RS^T^, GMI1000, CQPS-1, and *R. solanacearum* LMG 2299^T^ tested negative. Although the tested strains shared several phenotypic features, RS^T^, GMI1000, and CQPS-1 had unique characteristics. Strains of phylotype I tested negative for the use of dextrin, α-D-glucose, 3-methyl glucose, L-aspartic acid, L-glutamic acid, pectin, citric acid, L-malic acid, tween 40, and acetic acid. However, *R. pseudosolanacearum* LMG 9673^T^ and *R. solanacearum* LMG 2299^T^ could use these carbon sources (Supplementary Table 5).

The conserved fatty acids present in all tested strains were C_14:0_, C_16:0_, C_18:0_, cyclo-C_17:0_, C_18:1_ 2-OH, summed feature 2 (C_14:0_ 3-OH/C_16:1_ iso I), summed feature 3 (C_16:1_ *ω*7*c* and/or C_16:1_ *ω*6*c*), and summed feature 8 (C_18:1_ *ω*7*c* and/or C_18:1_ *ω*6*c*). The major fatty acids of strains RS^T^, GMI1000, and CQPS-1 were C_16:0_, summed feature 3 (C_16:1_ *ω*7*c* and/or C_16:1_ *ω*6*c*), and summed feature 8 (C_18:1_ *ω*7*c* and/or C_18:1_ *ω*6*c*), which were closely related to those of *R. pseudosolanacearum* LMG 9673^T^ and *R. solanacearum* LMG 2299^T^ (Table 3). The major fatty acids of these strains were similar to three recently proposed strains *R. wenshanensis* 56D2^T^ (Lu et al., 2022a), *R. chuxiongensis* 21YRMH01-3^T^, and *R. mojiangensis* 21MJYT02-10^T^ from our laboratory (Lu et al., 2023). The main fatty acids in tested phylotype I strains were slightly different from those of *R. soli* 21MJYT02-11^T^, which included the major fatty acids of C_16:0_, cyclo-C_17:0_, and summed features 3 (Lu et al., 2023).

**Table 3:** Major fatty acids of strain RS^T^ and its closely related strains in the genus *Ralstonia*. Strain: 1. RS^T^; 2. GMI1000; 3. CQPS-1; 4. *R. pseudosolanacearum* LMG 9673^T^; 5. *R. solanacearum* LMG 2299^T^; 6. *R. syzygii* subsp. *syzygii* LMG 10661^T^; 7. *R. syzygii* subsp. *celebesensis* LMG 27706^T^; 8. *R. syzygii* subsp. *indonesiensis* LMG 27703^T^.

Fatty acids	1	2	3	4	5	6	7	8
**Saturated**:								
C_14:0_	4.28 ± 0.26	3.42 ± 0.03	3.39 ± 0.04	5.03 ± 0.22	5.05 ± 0.34	1.88 ± 0.42	6.55 ± 0.56	4.74 ± 0.12
C_16:0_	30.99 ± 2.98	24.26 ± 1.33	25.30 ± 0.28	22.45 ± 1.59	29.85 ± 2.28	38.71 ± 1.85	34.63 ± 1.20	27.25 ± 0.93
C_18:0_	2.03 ± 0.97	8.97 ± 1.29	9.32 ± 0.16	3.21 ± 1.93	3.16 ± 1.28	14.09 ± 1.21	5.92 ± 1.78	1.76 ± 1.64
Cyclo-C_17:0_	4.57 ± 1.18	2.15 ± 0.19	2.10 ± 0.11	2.84 ± 1.51	7.11 ± 1.29	7.51 ± 0.36	15.70 ± 1.99	3.14 ± 0.57
**Unsaturated:**								
18:1 *ω*9*c*	4.54 ± 0.84	2.43 ± 1.03	2.09 ± 0.52	2.82 ± 0.63	3.97 ± 0.09	–	–	2.69 ± 1.18
**Hydroxy:**								
iso-C_11:0_ 3-OH	1.90 ± 0.42	TR	TR	1.04 ± 0.32	TR	–	–	1.00 ± 0.02
C_16:0_ 2-OH	1.19 ± 0.16	–	–	TR	1.31 ± 0.31	0.46 ± 0.08	2.01 ± 0.18	TR
C_16:1_ 2-OH	3.39 ± 0.59	TR	TR	3.71 ± 1.88	TR	0.84 ± 0.12	4.65 ± 0.67	1.46 ± 0.05
C_18:1_ 2-OH	5.30 ± 0.36	4.23 ± 0.19	4.34 ± 0.01	4.39 ± 0.53	3.62 ± 0.92	3.15 ± 0.24	4.28 ± 0.65	3.05 ± 0.54
**Summed features** ^**a** ^**:**								
2	7.89 ± 0.79	6.99 ± 0.25	6.34 ± 0.21	8.40 ± 1.05	10.16 ± 0.59	5.96 ± 0.35	15.36 ± 1.79	9.61 ± 0.18
3	19.12 ± 0.94	22.84 ± 0.82	21.10 ± 0.48	24.76 ± 3.26	20.76 ± 0.22	2.74 ± 0.08	5.19 ± 0.17	28.77 ± 2.07
8	13.70 ± 1.59	19.30 ± 1.31	21.05 ± 0.48	18.53 ± 0.98	11.70 ± 2.23	4.38 ± 0.43	5.72 ± 0.56	13.62 ± 1.33

The data are expressed as percentages of total fatty acids. TR, trace amount (less than 1.0% of the total); −, not detected.

a Summed features are fatty acids that cannot be resolved reliably from another fatty acid using the chromatographic conditions chosen. The MIDI system groups these fatty acids together as one feature with a single percentage of the total. Summed feature 2 comprises C_14:0_ 3-OH/C_16:1_ iso I. Summed feature 3 comprises C_16:1_ *ω*7*c* and/or C_16:1_ *ω*6*c*. Summed feature 8 comprises C_18:1_ *ω*7*c* and/or C_18:1_ *ω*6*c*.

The polar lipids analysis showed that strain RS^T^ contained phosphatidylethanolamine, diphosphatidylglycerol, phosphatidylglycerol, and an unidentified aminophospholipid (Supplementary Figure 4). Data from this study and our recent reports suggest that the major polar lipids of *Ralstonia* species are phosphatidylethanolamine and diphosphatidylglycerol (Lu et al., 2022a, 2023). In addition, isoprenoid quinone analysis showed that strain RS^T^ contained ubiquinone Q-7 (17.87%) and Q-8 (82.13%). Data from this study and our recent reports also indicated that Q-8 is the major respiratory quinone in *Ralstonia* species (Lu et al., 2022a, 2023).

## 4. Conclusion

Based on the above polyphasic analysis, we concluded that the *Ralstonia* strains could be phylogenetically separated into two main clusters: non-pathogenic and plant-pathogenic bacteria. The current *R. solanacearum* phylotype I strain RS^T^ and its close relatives represent a novel species in the genus *Ralstonia*, for which the name *Ralstonia nicotianae* sp. nov. is proposed.

## 5. Description of *Ralstonia nicotianae* sp. nov.

*Ralstonia nicotianae* (ni.co.ti.a’nae. N.L. gen. fem. n. *nicotianae*, of *Nicotiana*, the tobacco plant, from which the type strain (RS^T^) was isolated).

Cells are Gram-negative, aerobic, no-spore, motile with polar flagella, rod-shape, 1.60–3.60 μm long, and 0.68–1.44 μm wide (avg. 2.40 ± 0.52 μm × 0.93 ± 0.18 μm; *n* = 46). They can grow on LA, NA, PDA, TSA, CPG, TZC, and MacConkey agars, with better growth on CPG and TZC media. Colonies on TSA plates after incubation for 2 days at 28°C are circular, light yellow and convex, with a smooth surface, and 1.5 mm in diameter. Cells grow at 15–40°C (optimum, 25–35°C), 0–5% NaCl (w/v) (optimum, 0–2%), and at pH 5.0–10.0 (optimum, pH 7.0–8.0). Cells are positive for catalase and oxidase activities. In API 20NE assays, cells are positive for potassium nitrate reduction; esculin hydrolysis; and glucose, potassium gluconate, malate, and trisodium citrate assimilation; and negative for indole production; glucose fermentation; arginine dihydrolase; urease; β-galactosidase activities; gelatin hydrolysis; and arabinose, mannose, maltose and adipic acid assimilation. In the API ZYM analysis, strains are positive for enzymatic activities of leucine arylamidase and acid phosphatase; and negative for esterase lipase, lipase, valine arylamidase, cystine arylamidase, trypsin, α-chymotrypsin, naphthol-AS-BI-phosphohydrolase, α-galactosidase, β-glucuronidase, α-glucosidase, β-glucosidase, N-acetyl-β-glucosaminidase, α-mannosidase, and α-fucosidase activities. In the Biolog GEN III analysis, strains were positive for the utilization of D-galacturonic acid, L-galactonic acid lactone, D-glucuronic acid, glucuronamide, and D-fructose-6-PO_4_; and were resistant to rifamycin SV, lincomycin, vancomycin, tetrazolium violet, tetrazolium blue, and potassium tellurite. The primary fatty acids of RS^T^ are saturated fatty acid C_16:0_, summed feature 3 (C_16:1_ *ω*7*c* and/or C_16:1_ *ω*6*c*), and summed feature 8 (C_18:1_ *ω*7*c* and/or C_18:1_ *ω*6*c*). The polar lipid profile of strain RS^T^ consists of phosphatidylethanolamine, diphosphatidylglycerol, phosphatidylglycerol, and an unidentified aminophospholipid. Phosphatidylethanolamine is the major polar lipid. The strain contains ubiquinones Q-7 (17.87%) and Q-8 (82.13%).

The type strain, RS^T^ (= GDMCC 1.3533^T^ = JCM 35814^T^), was isolated from a bacterial wilt of flue-cured tobacco sampled in 2018 from Yuxi, Yunnan, China. The genomic DNA G + C content of type strain RS^T^ is 67.1 mol%. The GenBank accession number for the 16S rRNA gene sequence of the type strain is ON797093, and the genome sequence accession numbers are CP046674 and CP046675.

## Data availability statement

The datasets presented in this study can be found in online repositories. The names of the repository/repositories and accession number(s) can be found below: The GenBank accession numbers for the chromosome, megaplasmid and 16S rRNA gene sequences of strain RS^T^ are CP046674.1, CP046675.1, and ON797093.1, respectively.

## Author contributions

J-YL, H-ML, Y-QZ, and C-HL designed the research and project outline. J-YL collected the tobacco samples. J-YL, ZC, and H-LW performed the deposition and polyphasic taxonomy. J-YL, J-FZ, C-PZ, S-YL, and C-HL conducted the genome analysis. J-YL, C-HL, and Y-QZ drafted the manuscript. All authors read and approved the final manuscript.

## Funding

This work was supported by the Yunnan Fundamental Research Projects (202001BA0000-091 and 202201AT071023), the Yuxi Normal College Student Innovation and Entrepreneurship Training Program (2021A056), the Yunnan Provincial Tobacco Monopoly Bureau Projects (2020530000241013 and 2018530000241006), and the National Natural Science Foundation of China (32260702).

## Conflict of interest

The authors declare that the research was conducted in the absence of any commercial or financial relationships that could be construed as a potential conflict of interest.

## Publisher’s note

All claims expressed in this article are solely those of the authors and do not necessarily represent those of their affiliated organizations, or those of the publisher, the editors and the reviewers. Any product that may be evaluated in this article, or claim that may be made by its manufacturer, is not guaranteed or endorsed by the publisher.

## Footnotes

^1^ https://lpsn.dsmz.de/

^2^ https://www.ezbiocloud.net/

^3^ www.ncbi.nlm.nih.gov/genbank

^4^http://www.ebi.ac.uk/genomes/plasmid.html, last accessed July 8, 2016

## Abbreviations

ANI: average nucleotide identity
betalactone: beta-lactone-containing protease inhibitor
BGI: Beijing Genomics Institute
COG: cluster of orthologous groups
CPG: casamino acid peptone glucose
*egl*: endoglucanase
GDMCC: Guangdong Microbial Culture Collection Center
GTDB: Genome Taxonomy Database
hserlactone: homoserine lactone
dDDH: digital DNA–DNA hybridization
ITS: internal transcribed spacer region
JCM: Japan Collection of Microorganisms
LA: Luria-Bertani agar
ME: minimum-evolutions
ML: maximum-likelihood
NA: nutrient agar
NJ: neighbor-joining
NRPS: non-ribosomal peptide synthetase
PDA: potato dextrose agar
PDCs: pyruvate decarboxylases
QS: quorum sensing
RiPP-like: ribosomally synthesized and post-translationally modified peptide product-like
rRNA: ribosomal RNA
sRNA: small RNA
T1PKS: type I polyketide synthase
tRNA: transfer RNA
TSA: trypticase soy agar
TYGS: the type (strain) genome server
TZC: Kelman’s tetrazolium chloride
