# Supplementary material for: Proposal to classify Ralstonia solanacearum phylotype I strains as Ralstonia nicotianae sp. nov., and a genomic comparison between members of the genus Ralstonia
Source: Front Microbiol. 2023 Mar 22;14:1135872. doi: 10.3389/fmicb.2023.1135872 (PMC10073495; doi:10.3389/fmicb.2023.1135872)
Supplement: Supplementary file 1 [file Data_Sheet_1.docx]

**SUPPLEMENTARY INFORMATION**

**Supplementary Figure 1** A neighbor-joining phylogenetic tree based on 16S rRNA sequences (1406 bp) showing the relationship between strain RS^T^ with other type species within the genus *Ralstonia*. The tree was developed using MEGA v11.0 software. Bootstrap values (>50) are shown at branch nodes as percentages of 1000 replications. ^T^ indicates the type strain. Strain RS^T^ is highlighted in bold. *Cupriavidus necator* N-1^T^ is used as an outgroup. The NCBI accession numbers are shown in parentheses. Bar, 0.005 substitutions per nucleotide position.

**Supplementary Figure 2** The maximum-parsimony phylogenetic tree based on 16S rRNA sequences (1406 bp) showing the relationship between strain RS^T^ and type species in the genus *Ralstonia*. The tree was developed employing MEGA v11.0 software. Bootstrap values (≥50) are shown at branching points as percentages of 1000 replicates. ^T^ indicates the type strain. Strain RS^T^ is highlighted in bold. *Cupriavidus necator* N-1^T^ is used as an outgroup. The GenBank accession numbers are shown in parentheses.

**Supplementary Figure 3** The phylogenomic tree showing the relationship between *Ralstonia* *nicotianae* strains and their closely related strains in the genus *Ralstonia*. The phylogenomic tree was constructed using the Type (Strain) Genome Server (Meier-Kolthoff and Göker, 2019). The numbers above branches are the Genome BLAST Distance Phylogeny approach (GBDP) pseudo-bootstrap support values > 50% from 100 replicates. ^T^ indicates the type strain. Strains belonging to *R. nicotianae* are shown in bold. *Cupriavidus necator* N-1^T^ is used as an outgroup. The genome assembly number for each strain is shown in parentheses. Bar, 0.02 changes per nucleotide position.

**Supplementary Figure 4** Two-dimensional TLC plates of polar lipids were extracted from strain RS^T^. The plate was sprayed with 10% (v/v) molybdophosphoric acid to show all polar lipids. DPG stands for diphosphatidylglycerol; PG stands for phosphatidylglycerol; PE stands for phosphatidylethanolamine; APL stands for unidentified aminophospholipid.

Supplementary Table 1 Genome comparisons between strains RS^T^ and *R. pseudosolanacearum* LMG 9673^T^, and their close strains with FastANI values >95% with strain RS^T^.

| Strain name | Assembly number | Classification | |  | RS^T^ | | | |  | LMG 9673^T^ | | | |
| --- | --- | --- | --- | --- | --- | --- | --- | --- | --- | --- | --- | --- | --- |
|  |  | Current | Proposed |  | FastANI^a^ | DDH1^b^ | DDH2^b^ | DDH3^b^ |  | FastANI^a^ | DDH1^b^ | DDH2^b^ | DDH3^b^ |
| RS^T^ | GCA_018243235.1 | *R. pseudosolanacearum* | *R. nicotianae* |  | **100.00** | **100.00** | **100.00** | **100.00** |  | 95.97 | 74.90 | 66.20 | 75.80 |
| Y45 | GCA_000223115.2 | *R. solanacearum* | *R. nicotianae* |  | **99.23** | **92.20** | **93.50** | **94.60** |  | 95.92 | 72.00 | 66.40 | 73.40 |
| Tb04 | GCA_023130705.1 | *R. pseudosolanacearum* | *R. nicotianae* |  | **99.15** | **91.90** | **93.10** | **94.30** |  | 95.90 | 73.80 | 65.80 | 74.80 |
| RD15 | GCA_001854265.1 | *R. solanacearum* | *R. nicotianae* |  | **99.15** | **93.40** | **92.90** | **95.40** |  | 96.00 | 74.50 | 66.20 | 75.50 |
| Cm01 | GCA_023130835.1 | *R. pseudosolanacearum* | *R. nicotianae* |  | **99.14** | **93.50** | **92.90** | **95.40** |  | 95.99 | 76.00 | 65.20 | 76.60 |
| Pn33 | GCA_023130755.1 | *R. pseudosolanacearum* | *R. nicotianae* |  | **99.15** | **91.60** | **92.80** | **94.10** |  | 96.01 | 74.20 | 65.80 | 75.10 |
| PSS190 | GCA_001870825.1 | *R. solanacearum* | *R. nicotianae* |  | **99.13** | **89.30** | **92.60** | **92.40** |  | 96.05 | 72.60 | 65.80 | 73.80 |
| 362200 | GCA_015999365.1 | *R. solanacearum* | *R. nicotianae* |  | **99.12** | **92.30** | **92.60** | **94.60** |  | 95.98 | 73.90 | 65.80 | 74.80 |
| SL2330 | GCA_003515225.1 | *R. solanacearum* | *R. nicotianae* |  | **99.12** | **94.90** | **92.50** | **96.40** |  | 95.89 | 76.70 | 65.20 | 77.20 |
| B2 | GCA_011290405.1 | *R. solanacearum* | *R. nicotianae* |  | **99.14** | **91.80** | **92.50** | **94.20** |  | 95.91 | 75.60 | 64.80 | 76.10 |
| Bg07 | GCA_003256445.1 | *R. pseudosolanacearum* | *R. nicotianae* |  | **99.12** | **91.20** | **92.40** | **93.70** |  | 95.99 | 73.60 | 65.80 | 74.60 |
| CIP264_UW503 | GCA_023075905.1 | *R. pseudosolanacearum* | *R. nicotianae* |  | **99.02** | **84.10** | **92.40** | **88.30** |  | 96.11 | 71.20 | 66.50 | 72.70 |
| PeaFJ1 | GCA_023518395.1 | *R. pseudosolanacearum* | *R. nicotianae* |  | **99.11** | **92.90** | **92.40** | **95.00** |  | 95.95 | 74.60 | 65.40 | 75.40 |
| FQY_4 | GCA_000348545.1 | *R. solanacearum* | *R. nicotianae* |  | **98.94** | **55.30** | **92.30** | **61.70** |  | 95.78 | 44.60 | 66.90 | 47.60 |
| Pe_4 | GCA_012062655.1 | *R. solanacearum* | *R. nicotianae* |  | **99.03** | **92.30** | **92.30** | **94.50** |  | 95.98 | 75.90 | 65.50 | 76.50 |
| KACC10709 | GCA_001708525.1 | *R. solanacearum* | *R. nicotianae* |  | **98.96** | **89.40** | **92.20** | **92.40** |  | 95.92 | 73.60 | 65.70 | 74.60 |
| SL3103 | GCA_003515205.1 | *R. solanacearum* | *R. nicotianae* |  | **98.97** | **90.00** | **92.20** | **92.80** |  | 95.88 | 74.10 | 65.60 | 75.00 |
| T25 | GCA_003515305.1 | *R. solanacearum* | *R. nicotianae* |  | **99.11** | **95.10** | **92.20** | **96.50** |  | 95.87 | 76.20 | 65.00 | 76.70 |
| SL3755 | GCA_003515345.1 | *R. solanacearum* | *R. nicotianae* |  | **99.09** | **94.70** | **92.20** | **96.20** |  | 95.85 | 76.40 | 64.90 | 76.80 |
| T2C-Rasto | GCA_019737115.1 | *R. pseudosolanacearum* | *R. nicotianae* |  | **99.04** | **90.00** | **92.20** | **92.90** |  | 95.96 | 75.30 | 65.40 | 76.00 |
| To_53 | GCA_012062725.1 | *R. solanacearum* | *R. nicotianae* |  | **99.02** | **91.10** | **92.10** | **93.60** |  | 96.10 | 74.20 | 66.10 | 75.20 |
| To_22 | GCA_012062775.1 | *R. solanacearum* | *R. nicotianae* |  | **99.02** | **91.00** | **92.10** | **93.60** |  | 96.08 | 74.30 | 66.20 | 75.30 |
| Ec03 | GCA_023130775.1 | *R. pseudosolanacearum* | *R. nicotianae* |  | **99.05** | **91.40** | **92.10** | **93.90** |  | 95.91 | 76.20 | 65.30 | 76.70 |
| Ep08 | GCA_023130815.1 | *R. pseudosolanacearum* | *R. nicotianae* |  | **99.03** | **90.20** | **92.10** | **93.00** |  | 96.02 | 73.70 | 65.60 | 74.70 |
| P781 | GCA_001644865.1 | *R. solanacearum* | *R. nicotianae* |  | **99.00** | **92.60** | **92.00** | **94.70** |  | 95.83 | 77.10 | 65.30 | 77.50 |
| T42 | GCA_003515565.1 | *R. solanacearum* | *R. nicotianae* |  | **99.00** | **88.00** | **92.00** | **91.30** |  | 95.94 | 72.70 | 65.40 | 73.80 |
| PD:1419 | GCA_015912145.1 | *R. pseudosolanacearum* | *R. nicotianae* |  | **99.05** | **91.80** | **92.00** | **94.10** |  | 96.02 | 74.50 | 65.50 | 75.30 |
| Rs123_UW585 | GCA_023075675.1 | *R. solanacearum* | *R. nicotianae* |  | **99.03** | **90.90** | **92.00** | **93.50** |  | 95.94 | 76.30 | 65.40 | 76.90 |
| 10154 | GCA_008271845.1 | *R. solanacearum* | *R. nicotianae* |  | **98.97** | **91.50** | **91.90** | **93.90** |  | 95.93 | 76.70 | 65.60 | 77.20 |
| To_28 | GCA_012062765.1 | *R. solanacearum* | *R. nicotianae* |  | **99.01** | **92.00** | **91.90** | **94.30** |  | 96.04 | 76.10 | 65.60 | 76.70 |
| Pe_45 | GCA_012271415.1 | *R. solanacearum* | *R. nicotianae* |  | **99.04** | **91.00** | **91.90** | **93.50** |  | 95.99 | 75.60 | 65.40 | 76.30 |
| FJAT445.F1 | GCA_013306255.1 | *R. solanacearum* | *R. nicotianae* |  | **99.02** | **92.00** | **91.90** | **94.30** |  | 95.81 | 77.00 | 64.90 | 77.30 |
| PD:3205 | GCA_015911615.1 | *R. pseudosolanacearum* | *R. nicotianae* |  | **99.06** | **92.00** | **91.90** | **94.30** |  | 95.96 | 77.70 | 65.50 | 78.00 |
| CIP365_UW642 | GCA_023075535.1 | *R. pseudosolanacearum* | *R. nicotianae* |  | **99.02** | **89.50** | **91.90** | **92.50** |  | 95.96 | 76.30 | 65.50 | 76.90 |
| PSS4 | GCA_001876985.1 | *R. solanacearum* | *R. nicotianae* |  | **98.99** | **88.20** | **91.80** | **91.50** |  | 96.02 | 74.10 | 65.60 | 75.00 |
| RSCM | GCA_002894285.1 | *R. solanacearum* | *R. nicotianae* |  | **98.87** | **86.70** | **91.80** | **90.30** |  | 95.80 | 74.40 | 64.90 | 75.10 |
| T110 | GCA_003515465.1 | *R. solanacearum* | *R. nicotianae* |  | **99.08** | **95.10** | **91.80** | **96.40** |  | 95.82 | 77.00 | 64.70 | 77.30 |
| T523 | GCA_003595325.1 | *R. solanacearum* | *R. nicotianae* |  | **98.95** | **89.70** | **91.80** | **92.50** |  | 95.91 | 76.00 | 65.30 | 76.60 |
| Tg03 | GCA_003725665.1 | *R. pseudosolanacearum* | *R. nicotianae* |  | **98.99** | **89.50** | **91.80** | **92.40** |  | 95.98 | 74.10 | 65.30 | 74.90 |
| HA4-1 | GCA_003999715.1 | *R. solanacearum* | *R. nicotianae* |  | **99.13** | **90.50** | **91.80** | **93.10** |  | 95.92 | 72.90 | 65.40 | 73.90 |
| FJAT15340.F50 | GCA_013306375.1 | *R. solanacearum* | *R. nicotianae* |  | **98.94** | **89.60** | **91.80** | **92.50** |  | 95.86 | 76.00 | 65.30 | 76.50 |
| FJAT15340.F6 | GCA_013306395.1 | *R. solanacearum* | *R. nicotianae* |  | **98.94** | **89.60** | **91.80** | **92.50** |  | 95.86 | 76.00 | 65.30 | 76.50 |
| FJAT15340.F1 | GCA_013306415.1 | *R. solanacearum* | *R. nicotianae* |  | **98.94** | **89.60** | **91.80** | **92.50** |  | 95.86 | 76.00 | 65.30 | 76.50 |
| FJAT15304.F6 | GCA_013306435.1 | *R. solanacearum* | *R. nicotianae* |  | **98.94** | **89.60** | **91.80** | **92.50** |  | 95.86 | 76.00 | 65.30 | 76.50 |
| FJAT15304.F1 | GCA_013306455.1 | *R. solanacearum* | *R. nicotianae* |  | **98.94** | **89.60** | **91.80** | **92.50** |  | 95.86 | 76.00 | 65.30 | 76.50 |
| FJAT15304.F50 | GCA_013306475.1 | *R. solanacearum* | *R. nicotianae* |  | **98.94** | **89.60** | **91.80** | **92.50** |  | 95.86 | 76.00 | 65.30 | 76.50 |
| Rs-SY1 | GCA_018731965.1 | *R. solanacearum* | *R. nicotianae* |  | **98.98** | **90.20** | **91.80** | **92.90** |  | 95.89 | 76.00 | 65.20 | 76.60 |
| UW296 | GCA_023076985.1 | *R. solanacearum* | *R. nicotianae* |  | **99.00** | **90.80** | **91.80** | **93.40** |  | 95.87 | 75.40 | 65.60 | 76.10 |
|  | GCA_946480645.1 | *R. solanacearum* | *R. nicotianae* |  | **n/a** | **90.90** | **91.80** | **93.40** |  | n/a | 76.50 | 65.60 | 77.00 |
| PSS1308 | GCA_001870805.1 | *R. solanacearum* | *R. nicotianae* |  | **99.00** | **91.20** | **91.70** | **93.60** |  | 95.92 | 75.10 | 65.70 | 75.90 |
| Fm03 | GCA_003256405.1 | *R. pseudosolanacearum* | *R. nicotianae* |  | **98.93** | **92.00** | **91.70** | **94.20** |  | 95.94 | 76.70 | 65.30 | 77.20 |
| Cq01 | GCA_003256425.1 | *R. pseudosolanacearum* | *R. nicotianae* |  | **98.97** | **89.60** | **91.70** | **92.50** |  | 95.88 | 74.80 | 65.20 | 75.50 |
| SL3730 | GCA_003515365.1 | *R. solanacearum* | *R. nicotianae* |  | **99.00** | **91.50** | **91.70** | **93.90** |  | 95.90 | 76.40 | 65.20 | 76.90 |
| SL2729 | GCA_003515405.1 | *R. solanacearum* | *R. nicotianae* |  | **99.01** | **91.60** | **91.70** | **94.00** |  | 95.93 | 76.40 | 65.20 | 76.90 |
| Pe_2 | GCA_012062585.1 | *R. solanacearum* | *R. nicotianae* |  | **98.96** | **91.50** | **91.70** | **93.90** |  | 95.92 | 76.20 | 65.50 | 76.80 |
| Pe_9 | GCA_012062605.1 | *R. solanacearum* | *R. nicotianae* |  | **99.08** | **92.30** | **91.70** | **94.50** |  | 95.97 | 76.40 | 65.10 | 76.90 |
| Pe_30 | GCA_012271445.1 | *R. solanacearum* | *R. nicotianae* |  | **99.05** | **88.60** | **91.70** | **91.70** |  | 95.95 | 74.40 | 65.30 | 75.10 |
| FJAT445.F50 | GCA_013306235.1 | *R. solanacearum* | *R. nicotianae* |  | **99.04** | **92.00** | **91.70** | **94.30** |  | 95.78 | 77.00 | 64.90 | 77.30 |
| FJAT442.F50 | GCA_013306275.1 | *R. solanacearum* | *R. nicotianae* |  | **99.06** | **92.00** | **91.70** | **94.30** |  | 95.77 | 77.00 | 64.90 | 77.30 |
| FJAT442.F1 | GCA_013306295.1 | *R. solanacearum* | *R. nicotianae* |  | **99.06** | **92.00** | **91.70** | **94.30** |  | 95.77 | 77.00 | 64.90 | 77.30 |
| FJAT1452.F50 | GCA_013306845.1 | *R. solanacearum* | *R. nicotianae* |  | **99.06** | **92.00** | **91.70** | **94.30** |  | 95.77 | 77.00 | 64.90 | 77.30 |
| FJAT1452.F1 | GCA_013306875.1 | *R. solanacearum* | *R. nicotianae* |  | **99.06** | **92.00** | **91.70** | **94.30** |  | 95.77 | 77.00 | 64.90 | 77.30 |
| POPS1_UW379 | GCA_023076705.1 | *R. pseudosolanacearum* | *R. nicotianae* |  | **98.94** | **89.10** | **91.70** | **92.10** |  | 95.95 | 75.00 | 65.40 | 75.80 |
| SD54 | GCA_000430925.2 | *R. solanacearum* | *R. nicotianae* |  | **99.00** | **89.50** | **91.60** | **92.40** |  | 95.97 | 74.60 | 65.40 | 75.40 |
| PSS216 | GCA_001876975.1 | *R. solanacearum* | *R. nicotianae* |  | **98.93** | **89.80** | **91.60** | **92.60** |  | 95.85 | 73.90 | 65.40 | 74.80 |
| 10179 | GCA_008271855.1 | *R. solanacearum* | *R. nicotianae* |  | **98.99** | **91.10** | **91.60** | **93.60** |  | 95.85 | 76.20 | 65.50 | 76.80 |
| To_36 | GCA_012062745.1 | *R. solanacearum* | *R. nicotianae* |  | **99.08** | **91.50** | **91.60** | **93.80** |  | 95.96 | 75.90 | 65.10 | 76.40 |
| To_1 | GCA_012062825.1 | *R. solanacearum* | *R. nicotianae* |  | **99.02** | **90.20** | **91.60** | **92.90** |  | 95.89 | 74.80 | 65.20 | 75.60 |
| Pe_61 | GCA_012271315.1 | *R. solanacearum* | *R. nicotianae* |  | **98.96** | **90.00** | **91.60** | **92.80** |  | 95.89 | 75.50 | 65.40 | 76.20 |
| Pe_42 | GCA_012271435.1 | *R. solanacearum* | *R. nicotianae* |  | **99.05** | **91.40** | **91.60** | **93.80** |  | 95.94 | 75.80 | 65.10 | 76.40 |
| 28-12BR_UW352 | GCA_023076635.1 | *R. solanacearum* | *R. nicotianae* |  | **98.98** | **90.20** | **91.60** | **93.00** |  | 95.96 | 75.00 | 65.40 | 75.70 |
| Pp26 | GCA_023130735.1 | *R. pseudosolanacearum* | *R. nicotianae* |  | **99.06** | **91.20** | **91.60** | **93.70** |  | 95.88 | 75.60 | 65.10 | 76.20 |
| RS-N | GCA_024023015.1 | *R. solanacearum* | *R. nicotianae* |  | **98.99** | **89.50** | **91.60** | **92.40** |  | 95.93 | 76.10 | 65.10 | 76.60 |
| Rs-T02 | GCA_001484095.1 | *R. solanacearum* | *R. nicotianae* |  | **99.04** | **91.30** | **91.50** | **93.70** |  | 95.90 | 75.40 | 65.20 | 76.10 |
| T117 | GCA_003515245.1 | *R. solanacearum* | *R. nicotianae* |  | **99.00** | **92.00** | **91.50** | **94.20** |  | 95.86 | 76.70 | 64.80 | 77.10 |
| SL3822 | GCA_003515605.1 | *R. solanacearum* | *R. nicotianae* |  | **99.02** | **90.20** | **91.50** | **92.90** |  | 95.84 | 75.80 | 65.00 | 76.40 |
| 10180 | GCA_008271775.1 | *R. solanacearum* | *R. nicotianae* |  | **98.94** | **91.00** | **91.50** | **93.50** |  | 95.80 | 76.00 | 65.40 | 76.60 |
| Pe_56 | GCA_012271305.1 | *R. solanacearum* | *R. nicotianae* |  | **98.98** | **90.80** | **91.50** | **93.30** |  | 95.98 | 76.10 | 65.50 | 76.70 |
| Pe_51 | GCA_012271355.1 | *R. solanacearum* | *R. nicotianae* |  | **98.97** | **90.00** | **91.50** | **92.70** |  | 95.93 | 75.50 | 65.40 | 76.20 |
| Pe_49 | GCA_012271385.1 | *R. solanacearum* | *R. nicotianae* |  | **98.96** | **92.70** | **91.50** | **94.70** |  | 95.98 | 75.60 | 65.50 | 76.30 |
| Pe_26 | GCA_012271495.1 | *R. solanacearum* | *R. nicotianae* |  | **99.05** | **89.30** | **91.50** | **92.20** |  | 95.99 | 75.80 | 65.30 | 76.40 |
| FJAT1303.F1 | GCA_013306955.1 | *R. solanacearum* | *R. nicotianae* |  | **98.89** | **90.60** | **91.50** | **93.20** |  | 95.87 | 76.20 | 65.30 | 76.70 |
| MAFF 241647 | GCA_015098595.1 | *R. solanacearum* | *R. nicotianae* |  | **99.01** | **90.80** | **91.50** | **93.40** |  | 95.77 | 76.80 | 64.80 | 77.10 |
| SY1 | GCA_018540425.1 | *R. solanacearum* | *R. nicotianae* |  | **99.04** | **91.80** | **91.50** | **94.10** |  | 95.97 | 74.80 | 65.10 | 75.50 |
| SL1931 | GCA_022625045.1 | *R. pseudosolanacearum* | *R. nicotianae* |  | **98.95** | **88.50** | **91.50** | **91.70** |  | 95.91 | 75.50 | 65.00 | 76.00 |
| UW74 | GCA_023075215.1 | *R. solanacearum* | *R. nicotianae* |  | **99.02** | **91.70** | **91.50** | **94.00** |  | 96.04 | 74.70 | 65.50 | 75.50 |
| Pe_57 | GCA_012062545.1 | *R. solanacearum* | *R. nicotianae* |  | **99.01** | **91.60** | **91.40** | **94.00** |  | 95.85 | 75.00 | 65.60 | 75.80 |
| Pe_13 | GCA_012062595.1 | *R. solanacearum* | *R. nicotianae* |  | **99.00** | **90.20** | **91.40** | **92.90** |  | 95.99 | 75.20 | 65.30 | 75.90 |
| To_42 | GCA_012062685.1 | *R. solanacearum* | *R. nicotianae* |  | **98.99** | **91.40** | **91.40** | **93.70** |  | 95.96 | 75.80 | 65.10 | 76.30 |
| Pe_52 | GCA_012271325.1 | *R. solanacearum* | *R. nicotianae* |  | **98.93** | **90.60** | **91.40** | **93.20** |  | 95.91 | 75.90 | 65.40 | 76.50 |
| Pe_28 | GCA_012271485.1 | *R. solanacearum* | *R. nicotianae* |  | **98.99** | **89.10** | **91.40** | **92.10** |  | 95.94 | 75.90 | 65.20 | 76.40 |
| Pe_24 | GCA_012271545.1 | *R. solanacearum* | *R. nicotianae* |  | **98.95** | **89.70** | **91.40** | **92.50** |  | 95.92 | 74.50 | 65.20 | 75.20 |
| NCPPB:1579 | GCA_015912175.1 | *R. pseudosolanacearum* | *R. nicotianae* |  | **99.02** | **90.40** | **91.40** | **93.00** |  | 95.97 | 75.50 | 65.20 | 76.10 |
| P824 | GCA_003576625.1 | *R. solanacearum* | *R. nicotianae* |  | **99.01** | **88.20** | **91.30** | **91.40** |  | 95.80 | 73.70 | 65.20 | 74.50 |
| 204 | GCA_011290425.1 | *R. solanacearum* | *R. nicotianae* |  | **98.92** | **91.30** | **91.30** | **93.70** |  | 95.91 | 76.80 | 65.10 | 77.20 |
| Pe_1 | GCA_011420365.1 | *R. solanacearum* | *R. nicotianae* |  | **98.94** | **87.40** | **91.30** | **90.80** |  | 95.90 | 74.00 | 64.90 | 74.80 |
| Pe_3 | GCA_012062465.1 | *R. solanacearum* | *R. nicotianae* |  | **99.01** | **90.10** | **91.30** | **92.80** |  | 95.85 | 75.70 | 64.90 | 76.20 |
| Pe_39 | GCA_012062495.1 | *R. solanacearum* | *R. nicotianae* |  | **98.93** | **88.70** | **91.30** | **91.70** |  | 95.92 | 75.50 | 64.90 | 76.00 |
| Pe_27 | GCA_012062505.1 | *R. solanacearum* | *R. nicotianae* |  | **98.93** | **89.90** | **91.30** | **92.70** |  | 95.86 | 75.80 | 64.90 | 76.30 |
| To_7 | GCA_012062785.1 | *R. solanacearum* | *R. nicotianae* |  | **98.95** | **90.90** | **91.30** | **93.40** |  | 95.93 | 76.10 | 65.40 | 76.70 |
| FJAT15353.F8 | GCA_013306315.1 | *R. solanacearum* | *R. nicotianae* |  | **98.98** | **91.30** | **91.30** | **93.70** |  | 95.86 | 75.90 | 64.60 | 76.30 |
| FJAT15353.F50 | GCA_013306335.1 | *R. solanacearum* | *R. nicotianae* |  | **98.98** | **91.30** | **91.30** | **93.70** |  | 95.85 | 75.90 | 64.60 | 76.30 |
| FJAT15353.F1 | GCA_013306355.1 | *R. solanacearum* | *R. nicotianae* |  | **98.98** | **91.30** | **91.30** | **93.70** |  | 95.84 | 75.90 | 64.60 | 76.30 |
| FJAT1303.F8 | GCA_013306915.1 | *R. solanacearum* | *R. nicotianae* |  | **98.99** | **91.30** | **91.30** | **93.70** |  | 95.86 | 75.90 | 64.60 | 76.30 |
| FJAT1303.F50 | GCA_013306935.1 | *R. solanacearum* | *R. nicotianae* |  | **98.98** | **91.30** | **91.30** | **93.70** |  | 95.86 | 75.90 | 64.60 | 76.30 |
| P380 | GCA_021462455.1 | *R. solanacearum* | *R. nicotianae* |  | **98.89** | **91.50** | **91.30** | **93.80** |  | 95.91 | 74.50 | 65.50 | 75.30 |
| T25_UW811 | GCA_023074975.1 | *R. pseudosolanacearum* | *R. nicotianae* |  | **99.03** | **88.40** | **91.30** | **91.50** |  | 95.94 | 71.60 | 65.90 | 72.90 |
| B17_UW800 | GCA_023075035.1 | *R. pseudosolanacearum* | *R. nicotianae* |  | **98.86** | **91.40** | **91.30** | **93.80** |  | 95.70 | 75.50 | 65.80 | 76.20 |
| UW613 | GCA_023075595.1 | *R. solanacearum* | *R. nicotianae* |  | **98.95** | **90.70** | **91.30** | **93.20** |  | 95.86 | 74.90 | 65.70 | 75.70 |
| O12BS_UW407 | GCA_023076615.1 | *R. pseudosolanacearum* | *R. nicotianae* |  | **98.97** | **90.40** | **91.30** | **93.00** |  | 95.92 | 75.40 | 65.80 | 76.10 |
| Gg01 | GCA_023130765.1 | *R. pseudosolanacearum* | *R. nicotianae* |  | **99.03** | **90.70** | **91.30** | **93.20** |  | 95.99 | 74.50 | 65.30 | 75.30 |
| UW757 | GCA_001645725.1 | *R. solanacearum* | *R. nicotianae* |  | **98.92** | **91.70** | **91.20** | **94.00** |  | 95.93 | 76.00 | 65.50 | 76.60 |
| OE1-1 | GCA_001879565.1 | *R. solanacearum* | *R. nicotianae* |  | **98.98** | **92.00** | **91.20** | **94.20** |  | 95.93 | 75.40 | 65.40 | 76.10 |
| SL3300 | GCA_003515625.1 | *R. solanacearum* | *R. nicotianae* |  | **98.91** | **91.10** | **91.20** | **93.50** |  | 95.87 | 75.10 | 64.80 | 75.70 |
| Pe_15 | GCA_012062645.1 | *R. solanacearum* | *R. nicotianae* |  | **98.95** | **88.00** | **91.20** | **91.20** |  | 95.93 | 73.40 | 65.00 | 74.20 |
| To_63 | GCA_012062695.1 | *R. solanacearum* | *R. nicotianae* |  | **98.98** | **91.80** | **91.20** | **94.10** |  | 95.92 | 75.90 | 65.50 | 76.50 |
| MAFF 211491 | GCA_015098255.1 | *R. solanacearum* | *R. nicotianae* |  | **98.98** | **91.00** | **91.20** | **93.40** |  | 96.01 | 73.80 | 65.90 | 74.80 |
| MAFF 301560 | GCA_015098475.1 | *R. solanacearum* | *R. nicotianae* |  | **98.98** | **92.00** | **91.20** | **94.20** |  | 95.85 | 75.40 | 65.40 | 76.10 |
| PD 4138 | GCA_015911375.1 | *R. pseudosolanacearum* | *R. nicotianae* |  | **98.91** | **90.90** | **91.20** | **93.40** |  | 95.89 | 72.40 | 66.10 | 73.70 |
| FJ1003 | GCA_020923475.1 | *R. solanacearum* | *R. nicotianae* |  | **98.89** | **89.50** | **91.20** | **92.30** |  | 95.95 | 75.00 | 65.30 | 75.70 |
| B9_UW794 | GCA_023075075.1 | *R. solanacearum* | *R. nicotianae* |  | **98.95** | **90.00** | **91.20** | **92.70** |  | 95.91 | 73.40 | 65.70 | 74.40 |
| UW298 | GCA_023076935.1 | *R. solanacearum* | *R. nicotianae* |  | **98.98** | **92.20** | **91.20** | **94.30** |  | 95.90 | 76.60 | 64.90 | 77.00 |
| UW198 | GCA_023077395.1 | *R. pseudosolanacearum* | *R. nicotianae* |  | **98.96** | **90.80** | **91.20** | **93.30** |  | 95.93 | 74.80 | 65.50 | 75.60 |
| Tb18 | GCA_023130655.1 | *R. pseudosolanacearum* | *R. nicotianae* |  | **98.89** | **88.30** | **91.20** | **91.40** |  | 95.96 | 73.40 | 65.50 | 74.40 |
| YC40-M | GCA_001663415.1 | *R. solanacearum* | *R. nicotianae* |  | **98.94** | **86.80** | **91.10** | **90.30** |  | 95.80 | 73.40 | 64.80 | 74.20 |
| UTT-25 | GCA_002930085.2 | *R. solanacearum* | *R. nicotianae* |  | **98.87** | **91.00** | **91.10** | **93.40** |  | 95.64 | 73.60 | 65.60 | 74.60 |
| PD 3570 | GCA_015911505.1 | *R. pseudosolanacearum* | *R. nicotianae* |  | **98.90** | **91.10** | **91.10** | **93.50** |  | 95.96 | 72.70 | 66.10 | 73.90 |
| RS24 | GCA_018733955.1 | *R. solanacearum* | *R. nicotianae* |  | **98.98** | **88.90** | **91.10** | **91.90** |  | 95.86 | 74.80 | 65.00 | 75.50 |
| RS10 | GCA_018861195.1 | *R. solanacearum* | *R. nicotianae* |  | **98.98** | **89.90** | **91.10** | **92.60** |  | 95.87 | 75.80 | 64.70 | 76.20 |
| UW604 | GCA_023075615.1 | *R. solanacearum* | *R. nicotianae* |  | **98.92** | **90.80** | **91.10** | **93.30** |  | 95.93 | 75.50 | 65.50 | 76.20 |
| CIP266_UW505 | GCA_023075875.1 | *R. pseudosolanacearum* | *R. nicotianae* |  | **98.94** | **90.10** | **91.10** | **92.80** |  | 95.91 | 75.50 | 65.80 | 76.20 |
| Pt01 | GCA_023130685.1 | *R. pseudosolanacearum* | *R. nicotianae* |  | **98.96** | **89.60** | **91.10** | **92.40** |  | 96.06 | 75.00 | 65.50 | 75.80 |
| T60 | GCA_003515545.1 | *R. solanacearum* | *R. nicotianae* |  | **98.93** | **90.60** | **91.00** | **93.10** |  | 95.83 | 76.20 | 64.40 | 76.60 |
| 203 | GCA_011290445.1 | *R. solanacearum* | *R. nicotianae* |  | **98.94** | **91.30** | **91.00** | **93.70** |  | 95.86 | 76.80 | 65.10 | 77.20 |
| 202 | GCA_011290465.1 | *R. solanacearum* | *R. nicotianae* |  | **98.97** | **91.30** | **91.00** | **93.70** |  | 95.83 | 76.80 | 65.10 | 77.20 |
| FJAT454.F1 | GCA_013306095.1 | *R. solanacearum* | *R. nicotianae* |  | **98.95** | **90.90** | **91.00** | **93.30** |  | 95.87 | 74.30 | 65.00 | 75.00 |
| FJAT448.F50 | GCA_013306155.1 | *R. solanacearum* | *R. nicotianae* |  | **98.98** | **90.90** | **91.00** | **93.30** |  | 95.86 | 74.30 | 65.00 | 75.00 |
| FJAT448.F1 | GCA_013306205.1 | *R. solanacearum* | *R. nicotianae* |  | **98.95** | **90.90** | **91.00** | **93.30** |  | 95.85 | 74.30 | 65.00 | 75.00 |
| UW763 | GCA_014884725.1 | *R. solanacearum* | *R. nicotianae* |  | **98.86** | **91.50** | **91.00** | **93.80** |  | 95.86 | 76.00 | 65.40 | 76.60 |
| MAFF 241648 | GCA_015098755.1 | *R. solanacearum* | *R. nicotianae* |  | **98.97** | **89.40** | **91.00** | **92.20** |  | 95.88 | 72.30 | 65.90 | 73.50 |
| MAFF 311693 | GCA_015698385.1 | *R. solanacearum* | *R. nicotianae* |  | **98.87** | **89.10** | **91.00** | **92.00** |  | 95.93 | 72.90 | 65.80 | 74.00 |
| PD:3196 | GCA_015911645.1 | *R. pseudosolanacearum* | *R. nicotianae* |  | **98.86** | **90.50** | **91.00** | **93.10** |  | 95.72 | 72.20 | 66.10 | 73.50 |
| UW81 | GCA_023075015.1 | *R. solanacearum* | *R. nicotianae* |  | **98.90** | **90.90** | **91.00** | **93.30** |  | 95.83 | 75.70 | 65.50 | 76.40 |
| UW299 | GCA_023076895.1 | *R. solanacearum* | *R. nicotianae* |  | **98.87** | **89.90** | **91.00** | **92.60** |  | 95.84 | 73.60 | 65.60 | 74.60 |
| Bs715 | GCA_024802605.1 | *R. solanacearum* | *R. nicotianae* |  | **n/a** | **89.70** | **91.00** | **92.50** |  | n/a | 75.10 | 64.70 | 75.70 |
| Rs-10-244 | GCA_000671315.1 | *R. solanacearum* | *R. nicotianae* |  | **98.88** | **91.30** | **90.90** | **93.70** |  | 95.87 | 75.00 | 65.40 | 75.70 |
| CaRs-Mep | GCA_001855495.2 | *R. pseudosolanacearum* | *R. nicotianae* |  | **98.88** | **88.80** | **90.90** | **91.80** |  | 95.91 | 73.80 | 66.20 | 74.80 |
| FJAT-1458 | GCA_001887535.1 | *R. solanacearum* | *R. nicotianae* |  | **98.99** | **90.60** | **90.90** | **93.10** |  | 95.82 | 73.90 | 65.00 | 74.70 |
| EP1 | GCA_001891105.1 | *R. solanacearum* | *R. nicotianae* |  | **99.00** | **90.40** | **90.90** | **93.00** |  | 95.91 | 75.00 | 65.20 | 75.70 |
| SEPPX05 | GCA_002162015.1 | *R. solanacearum* | *R. nicotianae* |  | **98.79** | **85.40** | **90.90** | **89.20** |  | 95.84 | 72.40 | 65.50 | 73.50 |
| T78 | GCA_003515285.1 | *R. solanacearum* | *R. nicotianae* |  | **99.02** | **88.90** | **90.90** | **91.90** |  | 95.84 | 74.50 | 64.70 | 75.20 |
| SL3882 | GCA_003515585.1 | *R. solanacearum* | *R. nicotianae* |  | **98.95** | **90.60** | **90.90** | **93.10** |  | 95.85 | 76.20 | 64.40 | 76.60 |
| Pe_18 | GCA_012271525.1 | *R. solanacearum* | *R. nicotianae* |  | **98.97** | **88.30** | **90.90** | **91.40** |  | 95.95 | 73.80 | 65.00 | 74.60 |
| FJAT15252.F50 | GCA_013306495.1 | *R. solanacearum* | *R. nicotianae* |  | **99.01** | **90.60** | **90.90** | **93.10** |  | 95.87 | 73.90 | 65.00 | 74.70 |
| FJAT15249.F1 | GCA_013306575.1 | *R. solanacearum* | *R. nicotianae* |  | **99.01** | **90.60** | **90.90** | **93.10** |  | 95.85 | 73.90 | 65.00 | 74.70 |
| FJAT1458.F50 | GCA_013306785.1 | *R. solanacearum* | *R. nicotianae* |  | **99.01** | **90.60** | **90.90** | **93.10** |  | 95.86 | 73.90 | 65.00 | 74.70 |
| FJAT1463.F1 | GCA_013306805.1 | *R. solanacearum* | *R. nicotianae* |  | **99.00** | **90.60** | **90.90** | **93.10** |  | 95.86 | 73.90 | 65.00 | 74.70 |
| FJAT1458.F1 | GCA_013306825.1 | *R. solanacearum* | *R. nicotianae* |  | **99.00** | **90.60** | **90.90** | **93.10** |  | 95.86 | 73.90 | 65.00 | 74.70 |
| YQ | GCA_014041975.1 | *R. solanacearum* | *R. nicotianae* |  | **98.94** | **92.10** | **90.90** | **94.20** |  | 95.90 | 75.30 | 64.90 | 75.90 |
| FJAT454.F50-1 | GCA_014490805.1 | *R. solanacearum* | *R. nicotianae* |  | **98.96** | **90.90** | **90.90** | **93.30** |  | 95.89 | 74.20 | 65.00 | 75.00 |
| MAFF 211471 | GCA_015097935.1 | *R. solanacearum* | *R. nicotianae* |  | **98.92** | **88.50** | **90.90** | **91.50** |  | 95.82 | 74.40 | 64.50 | 75.00 |
| MAFF 211479 | GCA_015098135.1 | *R. solanacearum* | *R. nicotianae* |  | **98.95** | **90.40** | **90.90** | **93.00** |  | 95.99 | 72.40 | 66.00 | 73.60 |
| MAFF 211472 | GCA_015698345.1 | *R. solanacearum* | *R. nicotianae* |  | **98.94** | **88.50** | **90.90** | **91.50** |  | 95.79 | 74.40 | 64.50 | 75.00 |
| MAFF 211479 | GCA_015698365.1 | *R. solanacearum* | *R. nicotianae* |  | **98.95** | **90.40** | **90.90** | **93.00** |  | 95.96 | 72.40 | 66.00 | 73.60 |
| NCPPB 4029 | GCA_015910515.1 | *R. pseudosolanacearum* | *R. nicotianae* |  | **98.94** | **91.00** | **90.90** | **93.40** |  | 95.87 | 74.90 | 64.90 | 75.50 |
| PD:1256 | GCA_015912055.1 | *R. pseudosolanacearum* | *R. nicotianae* |  | **98.98** | **91.30** | **90.90** | **93.70** |  | 95.83 | 75.90 | 65.50 | 76.60 |
| NCPPB:790 | GCA_015912205.1 | *R. pseudosolanacearum* | *R. nicotianae* |  | **98.90** | **90.10** | **90.90** | **92.70** |  | 95.89 | 75.10 | 65.40 | 75.90 |
| Lallmahomed 30 | GCA_015912315.1 | *R. pseudosolanacearum* | *R. nicotianae* |  | **98.91** | **90.90** | **90.90** | **93.40** |  | 95.89 | 76.00 | 65.50 | 76.70 |
| Lallmahomed 16 | GCA_015912335.1 | *R. pseudosolanacearum* | *R. nicotianae* |  | **98.94** | **90.40** | **90.90** | **93.00** |  | 95.85 | 75.20 | 65.50 | 75.90 |
| Lallmahomed 54 | GCA_015912355.1 | *R. pseudosolanacearum* | *R. nicotianae* |  | **98.94** | **91.30** | **90.90** | **93.60** |  | 95.90 | 75.90 | 65.50 | 76.50 |
| Lallmahomed 7 | GCA_015912375.1 | *R. pseudosolanacearum* | *R. nicotianae* |  | **98.94** | **90.40** | **90.90** | **93.00** |  | 95.95 | 75.30 | 65.50 | 76.00 |
| FJAT-91 | GCA_000331875.1 | *R. solanacearum* | *R. nicotianae* |  | **98.88** | **87.00** | **90.80** | **90.40** |  | 95.59 | 68.60 | 65.30 | 70.10 |
| FJAT-1458 | GCA_000331895.1 | *R. solanacearum* | *R. nicotianae* |  | **98.99** | **83.50** | **90.80** | **87.60** |  | 95.71 | 64.40 | 65.50 | 66.40 |
| FJAT-91 | GCA_002155245.1 | *R. solanacearum* | *R. nicotianae* |  | **98.91** | **87.00** | **90.80** | **90.40** |  | 95.87 | 68.60 | 65.30 | 70.10 |
| FJAT15252.F1 | GCA_013306515.1 | *R. solanacearum* | *R. nicotianae* |  | **99.01** | **90.60** | **90.80** | **93.10** |  | 95.87 | 73.90 | 65.00 | 74.70 |
| FJAT15249.F50 | GCA_013306545.1 | *R. solanacearum* | *R. nicotianae* |  | **99.01** | **90.60** | **90.80** | **93.10** |  | 95.87 | 73.90 | 65.00 | 74.70 |
| FJAT1463.F50 | GCA_013306765.1 | *R. solanacearum* | *R. nicotianae* |  | **99.01** | **90.60** | **90.80** | **93.10** |  | 95.86 | 73.90 | 65.00 | 74.70 |
| NCPPB 253 | GCA_015910695.1 | *R. pseudosolanacearum* | *R. nicotianae* |  | **98.89** | **90.50** | **90.80** | **93.00** |  | 95.83 | 75.70 | 65.30 | 76.30 |
| Lallmahomed 66 | GCA_015912275.1 | *R. pseudosolanacearum* | *R. nicotianae* |  | **98.88** | **91.80** | **90.80** | **94.00** |  | 95.88 | 75.90 | 65.40 | 76.50 |
| Lallmahomed 59 | GCA_015912285.1 | *R. pseudosolanacearum* | *R. nicotianae* |  | **98.90** | **91.20** | **90.80** | **93.50** |  | 95.77 | 75.70 | 65.50 | 76.40 |
| B12_UW797 | GCA_023075095.1 | *R. solanacearum* | *R. nicotianae* |  | **98.93** | **87.70** | **90.80** | **90.90** |  | 95.90 | 73.90 | 65.50 | 74.80 |
| GMI1000 | GCA_000009125.1 | *R. solanacearum* | *R. nicotianae* |  | **98.91** | **90.70** | **90.70** | **93.20** |  | 95.91 | 75.60 | 65.60 | 76.30 |
| RS 476 | GCA_003595305.1 | *R. pseudosolanacearum* | *R. nicotianae* |  | **98.87** | **90.80** | **90.70** | **93.20** |  | 95.91 | 75.60 | 65.60 | 76.30 |
| VT0801 | GCA_008000395.1 | *R. pseudosolanacearum* | *R. nicotianae* |  | **98.76** | **82.90** | **90.70** | **87.10** |  | 95.41 | 64.60 | 65.40 | 66.50 |
| FJAT15244.F50 | GCA_013306655.1 | *R. solanacearum* | *R. nicotianae* |  | **98.96** | **89.70** | **90.70** | **92.40** |  | 95.88 | 75.40 | 65.00 | 76.00 |
| FJAT15244.F1 | GCA_013306745.1 | *R. solanacearum* | *R. nicotianae* |  | **98.95** | **89.70** | **90.70** | **92.40** |  | 95.87 | 75.40 | 65.00 | 76.00 |
| PD 3634 | GCA_015911495.1 | *R. pseudosolanacearum* | *R. nicotianae* |  | **98.89** | **91.80** | **90.70** | **93.90** |  | 95.94 | 75.90 | 65.40 | 76.50 |
| PD:7123 | GCA_015911755.1 | *R. pseudosolanacearum* | *R. nicotianae* |  | **98.80** | **89.00** | **90.70** | **91.90** |  | 95.90 | 75.30 | 65.20 | 76.00 |
| PD:1255 | GCA_015912195.1 | *R. pseudosolanacearum* | *R. nicotianae* |  | **98.91** | **91.80** | **90.70** | **94.00** |  | 95.92 | 75.90 | 65.50 | 76.50 |
| Lallmahomed 13 | GCA_015912385.1 | *R. pseudosolanacearum* | *R. nicotianae* |  | **98.87** | **91.70** | **90.70** | **93.90** |  | 95.84 | 75.80 | 65.40 | 76.40 |
| UQRS637_UW745 | GCA_023075155.1 | *R. solanacearum* | *R. nicotianae* |  | **98.89** | **91.50** | **90.70** | **93.80** |  | 95.88 | 75.20 | 65.70 | 76.00 |
| Rs-09-161 | GCA_000671335.1 | *R. solanacearum* | *R. nicotianae* |  | **98.77** | **89.90** | **90.60** | **92.60** |  | 95.90 | 75.40 | 65.40 | 76.10 |
| CQPS-1 | GCA_002220465.1 | *R. solanacearum* | *R. nicotianae* |  | **98.87** | **91.70** | **90.60** | **93.90** |  | 95.95 | 76.00 | 65.10 | 76.50 |
| FJAT91.F50 | GCA_013306015.1 | *R. solanacearum* | *R. nicotianae* |  | **98.89** | **91.90** | **90.60** | **94.00** |  | 95.84 | 76.00 | 64.60 | 76.40 |
| FJAT91-F1 | GCA_013375715.1 | *R. solanacearum* | *R. nicotianae* |  | **98.89** | **91.90** | **90.60** | **94.00** |  | 95.84 | 76.00 | 64.60 | 76.40 |
| FJAT15244-F8 | GCA_013704765.1 | *R. solanacearum* | *R. nicotianae* |  | **98.88** | **89.70** | **90.60** | **92.40** |  | 95.88 | 75.40 | 64.90 | 76.00 |
| Lallmahomed 72 | GCA_015912245.1 | *R. pseudosolanacearum* | *R. nicotianae* |  | **98.90** | **91.20** | **90.60** | **93.50** |  | 95.88 | 75.50 | 65.40 | 76.20 |
| FJAT91-F8 | GCA_013375735.1 | *R. solanacearum* | *R. nicotianae* |  | **98.93** | **91.90** | **90.50** | **94.00** |  | 95.84 | 76.00 | 64.50 | 76.40 |
| UW393 | GCA_023076675.1 | *R. solanacearum* | *R. nicotianae* |  | **98.95** | **90.70** | **90.50** | **93.20** |  | 95.86 | 75.40 | 65.60 | 76.10 |
| UW193 | GCA_023077445.1 | *R. solanacearum* | *R. nicotianae* |  | **98.85** | **90.90** | **90.50** | **93.30** |  | 95.81 | 74.90 | 65.70 | 75.70 |
| GRsMep | GCA_024460955.1 | *R. pseudosolanacearum* | *R. nicotianae* |  | **n/a** | **90.80** | **90.50** | **93.20** |  | n/a | 75.60 | 65.60 | 76.30 |
| YC45 | GCA_001267515.1 | *R. solanacearum* | *R. nicotianae* |  | **98.76** | **90.50** | **90.30** | **93.00** |  | 95.84 | 74.00 | 65.60 | 74.90 |
| PD 3278 | GCA_015910975.1 | *R. pseudosolanacearum* | *R. nicotianae* |  | **98.86** | **83.20** | **90.20** | **87.20** |  | 96.08 | 66.80 | 66.20 | 68.70 |
| FJAT-462 | GCA_001920895.2 | *R. solanacearum* | *R. nicotianae* |  | **97.89** | **63.40** | **89.80** | **69.50** |  | 94.74 | 49.70 | 64.00 | 52.30 |
| FJAT-452 | GCA_001920905.2 | *R. solanacearum* | *R. nicotianae* |  | **98.12** | **70.60** | **89.50** | **76.20** |  | 95.07 | 53.60 | 64.40 | 56.10 |
| FJAT-91 | GCA_001920885.2 | *R. solanacearum* | *R. nicotianae* |  | **97.60** | **59.30** | **88.70** | **65.30** |  | 94.44 | 44.20 | 63.90 | 46.80 |
| CFBP3059 | GCA_001644855.1 | *R. solanacearum* | *R. pseudosolanacearum* |  | 95.98 | 71.10 | 67.10 | 72.70 |  | **96.45** | **77.20** | **70.50** | **78.70** |
| CMR15 | GCA_000427195.1 | *R. solanacearum* | *R. pseudosolanacearum* |  | 96.01 | 76.60 | 66.80 | 77.40 |  | **97.20** | **78.30** | **76.30** | **80.80** |
| UW386 | GCA_006088755.1 | *R. solanacearum* | *R. pseudosolanacearum* |  | 95.99 | 70.60 | 66.70 | 72.20 |  | **96.46** | **76.40** | **70.80** | **78.10** |
| NCPPB 216 | GCA_015910735.1 | *R. pseudosolanacearum* | *R. pseudosolanacearum* |  | 96.03 | 66.30 | 66.70 | 68.40 |  | **98.25** | **83.40** | **85.90** | **86.80** |
| RUN2279 | GCA_014884705.1 | *R. solanacearum* | *R. pseudosolanacearum* |  | 95.95 | 71.30 | 66.50 | 72.80 |  | **99.15** | **97.80** | **94.30** | **98.50** |
| RUN2587_UW776 | GCA_023075115.1 | *R. pseudosolanacearum* | *R. pseudosolanacearum* |  | 96.06 | 75.20 | 66.40 | 76.10 |  | **99.29** | **96.90** | **94.70** | **97.90** |
| RUN2474 | GCA_014884685.1 | *R. solanacearum* | *R. pseudosolanacearum* |  | 95.85 | 73.00 | 66.30 | 74.30 |  | **99.19** | **97.30** | **94.90** | **98.20** |
| LMG 9673 | GCA_015910955.1 | *R. pseudosolanacearum* | *R. pseudosolanacearum* |  | 96.02 | 70.10 | 66.30 | 71.70 |  | **99.98** | **98.80** | **100.00** | **99.50** |
| NCPPB332_UW654 | GCA_023075415.1 | *R. pseudosolanacearum* | *R. pseudosolanacearum* |  | 95.96 | 74.30 | 66.30 | 75.30 |  | **98.48** | **91.10** | **86.50** | **92.90** |
| DGBBC1138_UW685 | GCA_023075375.1 | *R. pseudosolanacearum* | *R. pseudosolanacearum* |  | 95.97 | 75.80 | 66.20 | 76.60 |  | **97.25** | **84.70** | **76.00** | **86.10** |
| LMG 9673 | GCA_024925465.1 | *R. pseudosolanacearum* | *R. pseudosolanacearum* |  | 96.01 | 74.90 | 66.20 | 75.80 |  | **100.00** | **100.00** | **100.00** | **100.00** |
| LMG 9673 | GCA_919586305.1 | *R. pseudosolanacearum* | *R. pseudosolanacearum* |  | 96.01 | 75.70 | 66.20 | 76.50 |  | **100.00** | **100.00** | **100.00** | **100.00** |
| CIP296_UW472 | GCA_023076195.1 | *R. pseudosolanacearum* | *R. pseudosolanacearum* |  | 95.95 | 76.50 | 65.90 | 77.20 |  | **97.34** | **86.60** | **76.20** | **87.70** |

^a^ The FastANI values were tested using the FastANI v1.33 software (kmer 16, fragment length of 3000, and the minimal fraction is 0.2) (Jain et al., 2018) on the web server genome taxonomy database (GTDB) (Parks et al., 2021).

^b^ The dDDH values were calculated using the Genome-to-Genome Distance Calculator (Meier-Kolthoff et al., 2022). DDH1 = formula 1. DDH2 = formula 2. DDH3 = formula 3.

Supplementary Table 2 Genomic comparisons between RS^T^ and *R. pseudosolanacearum* LMG 9673^T^, and closely related strains of which shared FastANI values 93.00-98.00 with strain RS^T^.

| Strain | Source | Country | *R. nicotianae* RS^T^ | | | | | |  | *R. pseudosolanacearum* LMG 9673^T^ | | | | |
| --- | --- | --- | --- | --- | --- | --- | --- | --- | --- | --- | --- | --- | --- | --- |
|  |  |  | FastANI^a^ | ANIb^b^ | ANIm^b^ | DDH1^c^ | DDH2^c^ | DDH3^c^ | FastANI^a^ | ANIb^b^ | ANIm^b^ | DDH1^c^ | DDH2^c^ | DDH3^c^ |
| *R. pseudosolanacearum* CFBP3059 | Eggplant | Burkina Faso | 95.98 | 95.12 | 96.26 | 71.10 | 67.10 | 72.70 | **96.45** | **95.76** | **96.69** | **77.20** | **70.50** | **78.70** |
| *R. pseudosolanacearum* CMR15 | Tomato | Cameroon | 96.01 | 95.33 | 96.21 | 76.60 | 66.80 | 77.40 | **97.20** | **96.81** | **97.39** | **78.30** | **76.30** | **80.80** |
| *R. pseudosolanacearum* NCPPB 216 | n/a | Netherlands | 96.03 | 94.95 | 96.20 | 66.30 | 66.70 | 68.40 | **98.25** | **97.94** | **98.48** | **83.40** | **85.90** | **86.80** |
| *R. pseudosolanacearum* UW386 | Soil | Nigeria | 95.99 | 95.00 | 96.22 | 70.60 | 66.70 | 72.20 | **96.46** | **95.65** | **96.70** | **76.40** | **70.80** | **78.10** |
| *R. pseudosolanacearum* RUN2279 | Potato | Madagascar | 95.95 | 95.21 | 96.15 | 71.30 | 66.50 | 72.80 | **99.15** | **99.17** | **99.30** | **97.80** | **94.30** | **98.50** |
| *R. pseudosolanacearum* RUN2587_UW776 | Potato | Madagascar | 96.06 | 95.24 | 96.13 | 75.20 | 66.40 | 76.10 | **99.29** | **99.25** | **99.37** | **96.90** | **94.70** | **97.90** |
| *R. pseudosolanacearum* RUN2474 | Potato | Madagascar | 95.85 | 95.19 | 96.12 | 73.00 | 66.30 | 74.30 | **99.19** | **99.27** | **99.35** | **97.30** | **94.90** | **98.20** |
| *R. pseudosolanacearum* NCPPB332_UW654 | Potato | Zimbabwe | 95.96 | 95.24 | 96.14 | 74.30 | 66.30 | 75.30 | **98.48** | **98.23** | **98.53** | **91.10** | **86.50** | **92.90** |
| *R. pseudosolanacearum* DGBBC1138_UW685 | Potato | Guinea | 95.97 | 95.29 | 96.16 | 75.80 | 66.20 | 76.60 | **97.25** | **96.69** | **97.38** | **84.70** | **76.00** | **86.10** |
| *R. pseudosolanacearum* CIP296_UW472 | Potato | Nigeria | 95.95 | 95.29 | 96.14 | 76.50 | 65.90 | 77.20 | **97.34** | **96.83** | **97.44** | **86.60** | **76.20** | **87.70** |

^a^ The FastANI values were tested using the FastANI v1.33 software (kmer 16, fragment length of 3000, and the minimal fraction is 0.2) (Jain et al., 2018) on the web server genome taxonomy database (GTDB) (Parks et al., 2021).

^b^ The ANI (ANIb and ANIm) and overall G+C content values were calculated using the online service JSpeciesWS (Richter et al., 2015).

^c^ The dDDH values were predicted using the Genome-to-Genome Distance Calculator (Meier-Kolthoff et al., 2022). DDH1 = formula 1. DDH2 = formula 2. DDH3 = formula 3.

**Supplementary Table 3** The distribution of candidate T3Es in strain RS^T^ and other complete genome-sequenced strains from four phylotypes. Cells with green background mean that the effector is absent in all strains within the same species, and yellow background indicates the effector is conserved among strains within the same species. The white background indicates that the effector does not consistently exist in all strains from the same species, and the blue background suggests that the effector is conserved and specific for certain species. NO, unavailable information; OK, one copy of the effector is present in the strain; MT, several copies of the effector are present in the strain; PG, only a pseudogene copy of the effector is present in the strain.

| T3E | Phylotype | | | | | | | | | | | | | | | | |
| --- | --- | --- | --- | --- | --- | --- | --- | --- | --- | --- | --- | --- | --- | --- | --- | --- | --- |
|  | I | | | | | III | | | | | IIC | IIA | IIB | | IV | | |
|  | *Ralstonia nicotianae* | | | | | *R. pseudosolanacearum* | | | | | *R. solanacearum* | | | | *R. syzygii* | | |
|  | RS^T^ | GMI1000 | CQPS-1 | FQY_4 | EP1 | LMG 9673^T^ | CMR15 | RUN2279 | RUN2474 | UW386 | K60-1^T^ | CFBP2957 | Po82 | UW551 | PSI 7^T^ | Db01 | LLRS1 |
| RipS2 | NO | OK | NO | NO | NO | NO | OK | OK | OK | OK | NO | OK | OK | NO | OK | NO | NO |
| RipP2 | NO | OK | MT | OK | OK | OK | OK | OK | OK | OK | PG | NO | OK | NO | NO | NO | NO |
| RipS4 | NO | OK | OK | OK | NO | NO | OK | OK | NO | OK | OK | OK | OK | NO | OK | NO | NO |
| Hyp4 | NO | NO | NO | NO | NO | NO | OK | NO | NO | NO | NO | NO | NO | NO | OK | OK | NO |
| RipAE | OK | OK | OK | OK | OK | OK | OK | OK | OK | OK | PG | OK | OK | OK | OK | PG | OK |
| RipAA | OK | OK | NO | OK | OK | OK | OK | OK | OK | OK | PG | OK | OK | OK | OK | PG | OK |
| RipBI | NO | NO | NO | NO | NO | NO | NO | NO | NO | NO | PG | OK | OK | OK | NO | NO | NO |
| Hyp8 | NO | NO | NO | NO | NO | NO | NO | NO | NO | NO | NO | OK | OK | NO | NO | NO | NO |
| RipG5 | NO | OK | NO | OK | NO | OK | OK | NO | OK | NO | NO | OK | OK | OK | OK | OK | NO |
| RipBG | NO | NO | NO | NO | NO | NO | NO | NO | NO | NO | NO | NO | NO | NO | NO | NO | NO |
| RipF2 | NO | NO | NO | NO | NO | NO | NO | NO | NO | NO | OK | OK | OK | OK | NO | NO | OK |
| RipS3 | NO | OK | NO | NO | OK | NO | OK | NO | NO | NO | OK | OK | OK | FS | OK | OK | NO |
| RipBN | NO | NO | NO | NO | NO | NO | OK | NO | NO | NO | NO | NO | NO | NO | NO | NO | NO |
| RipBA | OK | PG | OK | OK | NO | NO | NO | NO | NO | NO | NO | NO | NO | NO | NO | NO | NO |
| RipAJ | OK | OK | OK | OK | OK | OK | OK | OK | OK | OK | OK | OK | OK | OK | OK | OK | OK |
| RipC1 | OK | OK | OK | OK | OK | PG | OK | PG | PG | PG | OK | OK | OK | OK | OK | OK | PG |
| RipAY | OK | OK | NO | OK | OK | OK | OK | OK | OK | OK | OK | OK | OK | OK | OK | OK | OK |
| RipG6 | OK | OK | NO | NO | OK | NO | OK | NO | NO | NO | NO | OK | OK | OK | OK | NO | OK |
| RipH2 | OK | OK | OK | NO | NO | OK | OK | NO | OK | NO | OK | OK | MT | OK | MT | NO | NO |
| Hyp17 | NO | NO | NO | NO | NO | NO | NO | NO | NO | NO | NO | NO | NO | NO | NO | NO | NO |
| RipT | NO | OK | OK | NO | OK | OK | NO | OK | NO | NO | NO | NO | NO | NO | OK | NO | NO |
| RipG7 | NO | OK | OK | NO | NO | NO | OK | OK | OK | OK | NO | OK | OK | OK | OK | NO | NO |
| RipAI | OK | OK | OK | OK | OK | OK | OK | OK | OK | OK | OK | OK | OK | OK | OK | OK | OK |
| RipN | OK | OK | OK | OK | OK | OK | OK | NO | OK | OK | OK | OK | OK | OK | OK | OK | OK |
| RipAG | NO | OK | NO | OK | NO | NO | NO | NO | NO | NO | NO | NO | NO | NO | OK | OK | OK |
| RipAF2 | NO | NO | NO | NO | NO | NO | NO | NO | NO | NO | NO | NO | NO | NO | NO | NO | NO |
| RipAZ2 | NO | NO | NO | NO | NO | NO | NO | NO | NO | NO | NO | NO | NO | NO | NO | NO | NO |
| RipL | OK | OK | OK | OK | OK | NO | OK | NO | NO | NO | OK | NO | OK | NO | FS | OK | OK |
| RipAC | OK | OK | PG | PG | OK | OK | OK | PG | PG | PG | OK | OK | OK | OK | OK | OK | OK |
| Hyp6 | OK | NO | OK | NO | OK | OK | MT | OK | NO | OK | NO | NO | NO | NO | NO | NO | NO |
| RipB | PG | OK | PG | OK | OK | OK | OK | OK | OK | OK | OK | OK | OK | OK | OK | OK | PG |
| RipAW | OK | OK | OK | OK | OK | OK | OK | NO | NO | NO | OK | OK | OK | NO | OK | NO | NO |
| Hyp18 | NO | NO | NO | NO | NO | OK | OK | OK | OK | OK | NO | NO | NO | NO | NO | OK | OK |
| RipAB | OK | OK | OK | OK | OK | OK | OK | OK | OK | OK | OK | OK | OK | OK | OK | OK | OK |
| RipA2 | NO | OK | OK | OK | NO | NO | OK | OK | NO | OK | OK | OK | OK | OK | OK | NO | OK |
| RipJ | OK | OK | OK | OK | OK | NO | NO | NO | NO | NO | NO | OK | OK | PG | NO | NO | NO |
| RipBB | NO | NO | NO | NO | NO | NO | NO | NO | NO | NO | NO | NO | NO | NO | OK | NO | OK |
| RipBH | NO | NO | NO | NO | NO | NO | NO | NO | NO | NO | NO | OK | OK | OK | OK | OK | NO |
| Hyp10 | NO | NO | NO | NO | NO | NO | NO | NO | NO | NO | NO | NO | PG | PG | NO | NO | NO |
| RipV2 | NO | NO | NO | NO | NO | NO | NO | NO | NO | NO | NO | OK | FS | OK | OK | OK | OK |
| RipAF1 | OK | OK | OK | OK | OK | NO | OK | NO | NO | NO | NO | OK | OK | NO | NO | NO | NO |
| RipH3 | NO | OK | NO | OK | OK | NO | OK | OK | NO | OK | NO | OK | OK | FS | OK | NO | OK |
| Hyp1 | NO | NO | NO | NO | NO | NO | NO | NO | NO | OK | OK | OK | OK | NO | OK | OK | OK |
| RipAD | OK | OK | PG | OK | OK | OK | OK | OK | OK | OK | OK | OK | OK | OK | OK | NO | OK |
| RipBC | NO | NO | NO | NO | NO | NO | NO | NO | NO | NO | NO | OK | OK | NO | NO | NO | NO |
| RipBD | NO | NO | PG | NO | NO | NO | NO | NO | NO | NO | OK | NO | OK | NO | NO | NO | NO |
| RipA1 | OK | OK | NO | OK | OK | NO | NO | NO | NO | NO | NO | NO | NO | NO | NO | NO | NO |
| Hyp2 | NO | NO | NO | NO | NO | NO | NO | NO | NO | NO | NO | NO | NO | NO | OK | OK | NO |
| RipTAL | OK | OK | OK | MT | OK | NO | PG | NO | NO | OK | NO | PG | NO | NO | OK | OK | OK |
| RipA4 | NO | OK | NO | OK | OK | NO | OK | OK | OK | NO | NO | OK | FS | FS | OK | OK | OK |
| RipC2 | OK | PG | OK | NO | NO | NO | PG | NO | NO | NO | NO | OK | NO | NO | NO | NO | NO |
| RipX | OK | OK | OK | OK | OK | OK | OK | OK | OK | OK | OK | OK | OK | OK | OK | OK | OK |
| RipP3 | NO | PG | NO | NO | NO | NO | NO | NO | NO | OK | NO | NO | NO | NO | NO | NO | NO |
| RipZ | OK | OK | OK | OK | OK | OK | OK | OK | OK | OK | OK | OK | OK | FS | OK | OK | OK |
| RipAK | OK | OK | OK | OK | OK | NO | NO | NO | NO | NO | NO | NO | NO | NO | NO | NO | NO |
| RipBJ | NO | OK | OK | NO | NO | NO | NO | NO | NO | NO | NO | NO | NO | NO | NO | OK | OK |
| RipU | OK | OK | OK | OK | OK | OK | OK | OK | OK | OK | OK | OK | OK | OK | OK | PG | OK |
| RipS1 | NO | OK | NO | NO | OK | OK | OK | NO | OK | NO | NO | OK | OK | OK | NO | NO | NO |
| RipG4 | NO | OK | NO | NO | NO | NO | OK | NO | NO | PG | NO | OK | OK | OK | NO | NO | NO |
| Hyp9 | NO | NO | NO | NO | NO | NO | NO | NO | NO | NO | PG | OK | OK | OK | NO | NO | NO |
| Hyp5 | NO | NO | NO | NO | NO | NO | NO | NO | NO | NO | NO | NO | NO | NO | OK | NO | NO |
| RipS6 | NO | OK | NO | OK | NO | NO | NO | NO | NO | NO | NO | NO | NO | NO | NO | NO | NO |
| RipAO | OK | OK | OK | OK | OK | OK | OK | OK | OK | OK | OK | OK | OK | OK | OK | OK | OK |
| RipAU | OK | OK | OK | OK | NO | OK | OK | OK | OK | OK | OK | OK | OK | NO | OK | OK | OK |
| RipBQ | NO | NO | NO | NO | NO | NO | NO | NO | NO | NO | NO | NO | NO | NO | NO | NO | NO |
| RipO2 | NO | NO | NO | NO | NO | NO | NO | NO | NO | NO | NO | NO | NO | NO | NO | NO | NO |
| RipAV | OK | OK | OK | OK | OK | OK | OK | NO | OK | OK | NO | OK | OK | OK | NO | NO | NO |
| RipM | OK | OK | NO | OK | OK | OK | OK | OK | OK | OK | OK | OK | FS | OK | OK | OK | OK |
| RipAX1 | NO | OK | OK | NO | OK | OK | NO | NO | NO | NO | NO | NO | OK | OK | NO | OK | NO |
| RipW | OK | OK | OK | OK | OK | PG | OK | OK | NO | OK | OK | OK | OK | OK | OK | PG | OK |
| RipA5 | NO | OK | OK | OK | NO | OK | FS | OK | OK | OK | OK | OK | MT | MT | OK | PG | OK |
| RipE2 | OK | NO | NO | NO | NO | OK | FS | OK | OK | OK | OK | OK | OK | OK | PG | OK | NO |
| RipY | OK | OK | NO | OK | OK | OK | OK | OK | OK | NO | OK | OK | OK | OK | OK | OK | OK |
| RipH4 | NO | NO | NO | NO | NO | NO | NO | NO | NO | NO | NO | NO | NO | NO | OK | OK | NO |
| RipG8 | NO | NO | NO | NO | NO | NO | OK | NO | NO | NO | NO | NO | NO | NO | NO | NO | NO |
| RipO1 | OK | OK | OK | OK | OK | OK | OK | OK | OK | OK | OK | OK | OK | OK | NO | NO | OK |
| Hyp11 | NO | NO | NO | NO | NO | NO | NO | NO | NO | NO | NO | NO | PG | PG | NO | NO | NO |
| RipS8 | OK | PG | NO | PG | OK | NO | NO | NO | NO | NO | NO | NO | NO | NO | OK | NO | NO |
| RipAS | OK | OK | OK | OK | OK | OK | OK | OK | OK | NO | NO | OK | OK | OK | PG | NO | OK |
| RipA3 | OK | OK | OK | NO | NO | OK | OK | OK | NO | OK | OK | OK | OK | NO | OK | NO | NO |
| RipG1 | NO | OK | OK | NO | NO | NO | OK | OK | OK | NO | NO | NO | NO | NO | MT | OK | NO |
| RipAZ1 | OK | OK | OK | OK | OK | OK | PG | PG | OK | OK | PG | OK | OK | NO | OK | OK | OK |
| Hyp14 | NO | NO | OK | NO | NO | NO | NO | NO | NO | NO | NO | NO | NO | NO | NO | NO | OK |
| RipBF | NO | NO | NO | NO | NO | NO | NO | NO | NO | NO | NO | NO | NO | NO | OK | PG | PG |
| RipBM | OK | PG | OK | OK | OK | OK | OK | OK | OK | OK | PG | OK | NO | NO | OK | NO | OK |
| RipBL | NO | NO | NO | OK | NO | NO | NO | NO | NO | NO | NO | NO | NO | NO | NO | NO | NO |
| RipBP | NO | NO | NO | NO | OK | NO | NO | NO | NO | NO | NO | NO | NO | NO | NO | NO | NO |
| RipBK | NO | NO | NO | OK | NO | NO | NO | NO | NO | NO | NO | NO | NO | NO | NO | NO | NO |
| RipG3 | NO | OK | NO | NO | NO | NO | OK | NO | NO | OK | OK | OK | OK | OK | OK | NO | OK |
| RipV1 | OK | OK | OK | OK | OK | NO | OK | NO | NO | OK | OK | OK | OK | OK | OK | OK | OK |
| RipAL | OK | NO | OK | OK | OK | OK | FS | OK | OK | OK | OK | OK | OK | OK | OK | OK | OK |
| RipE1 | OK | OK | OK | OK | OK | OK | OK | OK | OK | NO | OK | OK | OK | OK | MT | OK | OK |
| RipR | OK | OK | OK | OK | OK | OK | OK | OK | OK | OK | OK | OK | OK | OK | OK | OK | OK |
| Hyp13 | NO | NO | NO | NO | NO | NO | NO | NO | NO | NO | NO | NO | FS | NO | NO | NO | NO |
| RipS7 | NO | NO | NO | NO | NO | NO | NO | NO | NO | NO | NO | OK | OK | OK | OK | NO | OK |
| RipTPS | NO | OK | OK | NO | OK | OK | OK | NO | NO | NO | NO | OK | FS | OK | OK | NO | NO |
| RipI | OK | OK | OK | OK | OK | OK | OK | OK | OK | OK | OK | OK | OK | PG | OK | NO | OK |
| RipAX2 | MT | OK | NO | NO | NO | NO | NO | OK | OK | OK | NO | NO | NO | NO | NO | NO | OK |
| RipAH | OK | OK | OK | NO | NO | NO | NO | NO | NO | OK | NO | NO | NO | NO | NO | NO | NO |
| RipAQ | OK | OK | OK | OK | OK | OK | OK | OK | OK | OK | OK | OK | PG | OK | OK | OK | OK |
| RipAT | PG | PG | OK | OK | OK | NO | OK | NO | NO | NO | PG | OK | OK | OK | OK | OK | OK |
| RipAM | OK | OK | OK | OK | OK | OK | OK | OK | OK | OK | OK | OK | OK | OK | OK | OK | OK |
| RipAN | OK | OK | OK | OK | OK | OK | OK | OK | OK | OK | OK | OK | OK | OK | OK | PG | OK |
| RipBO | NO | OK | NO | NO | OK | NO | NO | NO | NO | NO | NO | NO | NO | NO | OK | NO | OK |
| RipQ | OK | OK | OK | OK | OK | OK | OK | OK | OK | OK | NO | OK | OK | PG | NO | NO | NO |
| RipG2 | OK | OK | OK | OK | OK | OK | OK | OK | OK | OK | NO | OK | OK | OK | OK | NO | NO |
| RipAR | NO | OK | NO | NO | NO | NO | OK | OK | OK | OK | NO | OK | OK | OK | OK | OK | OK |
| Hyp12 | NO | NO | NO | NO | NO | NO | NO | NO | NO | NO | NO | FS | PG | OK | NO | NO | NO |
| RipAP | OK | PG | OK | OK | OK | OK | OK | OK | OK | OK | OK | OK | OK | OK | NO | NO | NO |
| RipD | NO | OK | NO | OK | OK | OK | OK | OK | OK | OK | NO | OK | OK | OK | OK | OK | OK |
| RipK | NO | NO | NO | NO | NO | NO | NO | NO | NO | NO | PG | OK | NO | NO | NO | OK | OK |
| RipS5 | PG | OK | OK | OK | NO | OK | OK | NO | NO | NO | NO | PG | FS | NO | OK | OK | OK |
| RipP1 | NO | OK | OK | NO | OK | PG | NO | OK | PG | OK | NO | NO | NO | NO | NO | NO | NO |
| Hyp7 | OK | NO | NO | NO | OK | NO | OK | NO | NO | OK | NO | NO | NO | NO | NO | NO | NO |
| RipBE | NO | NO | OK | NO | NO | NO | NO | NO | NO | NO | NO | NO | NO | NO | NO | NO | NO |
| RipH1 | OK | OK | OK | OK | OK | OK | OK | OK | OK | OK | NO | OK | OK | OK | OK | NO | OK |
| Hyp3 | NO | NO | NO | NO | NO | NO | NO | NO | NO | NO | NO | NO | NO | NO | OK | OK | NO |
| RipF1 | OK | MT | OK | OK | OK | OK | MT | OK | OK | OK | NO | OK | OK | OK | OK | OK | NO |

Supplementary Table 4 *In silico* predictions of secondary metabolic biosynthetic gene clusters in strain RS^T^, its closely related strains, and type species in the genus *Ralstonia* using the bacterial antiSMASH database (Blin et al., 2021).

| Strain | Region | From | To | Size/kb | Type | Most similar known cluster | Similarity/% |
| --- | --- | --- | --- | --- | --- | --- | --- |
| *R. nicotinae* RS^T^ | Region 1.1 | 185,835 | 206,449 | 20.61 | hserlactone |  |  |
|  | Region 1.2 | 1,739,300 | 1,760,172 | 20.87 | terpene |  |  |
|  | Region 1.3 | 1,861,682 | 1,934,420 | 72.74 | NRPS,T1PKS | micacocidin | 100 |
|  | Region 1.4 | 2,982,296 | 3,002,940 | 20.64 | furan |  |  |
|  | Region 1.5 | 3,142,788 | 3,184,092 | 41.30 | arylpolyene | APE Vf | 10 |
|  | Region 2.1 | 114,476 | 198,437 | 83.96 | T1PKS,NRPS | ralsolamycin | 100 |
|  | Region 2.2 | 329,047 | 349,601 | 20.55 | hserlactone | obafluorin | 14 |
|  | Region 2.3 | 658,617 | 669,495 | 10.88 | RiPP-like |  |  |
|  | Region 2.4 | 1,101,651 | 1,150,161 | 48.51 | NRPS-like,NRPS |  |  |
|  | Region 2.5 | 1,552,249 | 1,607,596 | 55.35 | NRPS | marformycin A / marformycin B / marformycin C / marformycin D / marformycin E / marformycin F | 8 |
|  | Region 2.6 | 1,809,034 | 1,853,919 | 44.89 | arylpolyene | APE Vf | 35 |
|  | Region 2.7 | 1,855,984 | 1,909,714 | 53.73 | NRPS-like,siderophore | staphylobactin | 18 |
| *R. nicotianae* GMI1000 | Region 1.1 | 433,907 | 475,211 | 41.30 | arylpolyene | APE Vf | 10 |
|  | Region 1.2 | 615,573 | 636,397 | 20.82 | furan |  |  |
|  | Region 1.3 | 1,766,203 | 1,787,075 | 20.87 | terpene |  |  |
|  | Region 1.4 | 1,925,814 | 1,998,570 | 72.76 | NRPS,T1PKS | micacocidin | 100 |
|  | Region 1.5 | 3,527,741 | 3,548,355 | 20.61 | hserlactone |  |  |
|  | Region 2.1 | 177,438 | 226,588 | 49.15 | NRPS | lysocin | 14 |
|  | Region 2.2 | 442,366 | 487,251 | 44.89 | arylpolyene | APE Vf | 35 |
|  | Region 2.3 | 526,328 | 543,038 | 16.71 | siderophore | staphylobactin | 18 |
|  | Region 2.4 | 762,247 | 840,433 | 78.19 | T1PKS,NRPS | ralsolamycin | 100 |
|  | Region 2.5 | 969,956 | 990,510 | 20.55 | hserlactone | obafluorin | 14 |
|  | Region 2.6 | 1,306,527 | 1,317,405 | 10.88 | RiPP-like |  |  |
|  | Region 2.7 | 1,764,488 | 1,813,189 | 48.70 | NRPS-like,NRPS |  |  |
| *R. nicotianae* CQPS-1 | Region 1.1 | 794,760 | 815,695 | 20.94 | furan |  |  |
|  | Region 1.2 | 955,414 | 996,718 | 41.30 | arylpolyene | APE Vf | 10 |
|  | Region 1.3 | 1,651,362 | 1,671,976 | 20.61 | hserlactone |  |  |
|  | Region 1.4 | 3,302,796 | 3,323,668 | 20.87 | terpene |  |  |
|  | Region 1.5 | 3,428,356 | 3,498,388 | 70.03 | NRPS,T1PKS | micacocidin | 100 |
|  | Region 2.1 | 166,700 | 183,415 | 16.72 | siderophore | staphylobactin | 18 |
|  | Region 2.2 | 276,223 | 327,333 | 51.11 | NRPS-like,NRPS |  |  |
|  | Region 2.3 | 726,407 | 780,378 | 53.97 | NRPS | marformycin A / marformycin B / marformycin C / marformycin D / marformycin E / marformycin F | 12 |
|  | Region 2.4 | 991,984 | 1,036,869 | 44.89 | arylpolyene | APE Vf | 35 |
|  | Region 2.5 | 1,419,717 | 1,430,595 | 10.88 | RiPP-like |  |  |
|  | Region 2.6 | 1,723,493 | 1,744,047 | 20.55 | hserlactone | obafluorin | 14 |
|  | Region 2.7 | 1,876,208 | 1,961,095 | 84.89 | NRPS,T1PKS | ralsolamycin | 100 |
| *R. nicotianae* EP1 | Region 1.1 | 167,407 | 188,021 | 20.61 | hserlactone |  |  |
|  | Region 1.2 | 1,917,112 | 1,937,984 | 20.87 | terpene |  |  |
|  | Region 1.3 | 2,078,895 | 2,151,663 | 72.77 | NRPS,T1PKS | micacocidin | 100 |
|  | Region 1.4 | 3,282,888 | 3,303,823 | 20.94 | furan |  |  |
|  | Region 1.5 | 3,442,693 | 3,483,910 | 41.22 | arylpolyene | APE Vf | 10 |
|  | Region 2.1 | 270,295 | 324,046 | 53.75 | NRPS | marformycin A / marformycin B / marformycin C / marformycin D / marformycin E / marformycin F | 8 |
|  | Region 2.2 | 533,325 | 578,210 | 44.89 | arylpolyene | APE Vf | 35 |
|  | Region 2.3 | 590,628 | 644,172 | 53.54 | NRPS-like,siderophore | staphylobactin | 18 |
|  | Region 2.4 | 912,582 | 996,519 | 83.94 | T1PKS,NRPS | ralsolamycin | 100 |
|  | Region 2.5 | 1,145,514 | 1,166,068 | 20.55 | hserlactone | obafluorin | 14 |
|  | Region 2.6 | 1,476,087 | 1,486,965 | 10.88 | RiPP-like |  |  |
|  | Region 2.7 | 1,908,718 | 1,957,404 | 48.69 | NRPS-like,NRPS |  |  |
| *R. nicotianae* FQY_4 | Region 1.1 | 440,528 | 481,745 | 41.22 | arylpolyene | APE Vf | 10 |
|  | Region 1.2 | 597,186 | 618,121 | 20.94 | furan |  |  |
|  | Region 1.3 | 1,649,544 | 1,722,258 | 72.71 | NRPS,T1PKS | micacocidin | 100 |
|  | Region 1.4 | 1,817,822 | 1,838,694 | 20.87 | terpene |  |  |
|  | Region 1.5 | 3,525,330 | 3,545,944 | 20.61 | hserlactone |  |  |
|  | Region 2.1 | 127,508 | 181,948 | 54.44 | NRPS | marformycin A / marformycin B / marformycin C / marformycin D / marformycin E / marformycin F | 8 |
|  | Region 2.2 | 573,761 | 627,263 | 53.50 | siderophore,NRPS-like | staphylobactin | 18 |
|  | Region 2.3 | 639,687 | 684,572 | 44.89 | arylpolyene | APE Vf | 35 |
|  | Region 2.4 | 881,255 | 892,133 | 10.88 | RiPP-like |  |  |
|  | Region 2.5 | 1,219,588 | 1,240,142 | 20.55 | hserlactone | obafluorin | 14 |
|  | Region 2.6 | 1,368,123 | 1,427,364 | 59.24 | T1PKS,NRPS | ralsolamycin | 100 |
|  | Region 2.7 | 1,757,186 | 1,805,904 | 48.72 | NRPS-like,NRPS |  |  |
| *R. pseudosolanacearum* LMG 9673^T^ | Region 1.1 | 196,603 | 207,481 | 10.88 | RiPP-like |  |  |
|  | Region 1.2 | 794,014 | 814,334 | 20.32 | RRE-containing |  |  |
|  | Region 1.3 | 1,133,354 | 1,218,669 | 85.32 | NRPS,T1PKS | crocacin | 33 |
|  | Region 1.4 | 1,398,255 | 1,443,140 | 44.89 | arylpolyene | APE Vf | 35 |
|  | Region 1.5 | 1,473,584 | 1,490,300 | 16.72 | siderophore | staphylobactin | 18 |
|  | Region 1.6 | 1,624,919 | 1,693,713 | 68.79 | T1PKS,NRPS | ralsolamycin | 100 |
|  | Region 2.1 | 161,264 | 181,860 | 20.60 | hserlactone |  |  |
|  | Region 2.2 | 2,054,766 | 2,127,496 | 72.73 | NRPS,T1PKS | micacocidin | 100 |
|  | Region 2.3 | 2,235,120 | 2,255,992 | 20.87 | terpene |  |  |
|  | Region 2.4 | 2,995,734 | 3,042,201 | 46.47 | NRPS |  |  |
|  | Region 2.5 | 3,337,950 | 3,379,176 | 41.23 | arylpolyene | APE Vf | 10 |
| *R. pseudosolanacearum* CMR15 | Region 1.1 | 192,701 | 213,315 | 20.61 | hserlactone |  |  |
|  | Region 1.2 | 1,611,314 | 1,633,557 | 22.24 | LAP |  |  |
|  | Region 1.3 | 1,719,093 | 1,739,965 | 20.87 | terpene |  |  |
|  | Region 1.4 | 1,860,041 | 1,931,660 | 71.62 | T1PKS,NRPS | micacocidin | 100 |
|  | Region 1.5 | 2,842,871 | 2,889,338 | 46.47 | NRPS |  |  |
|  | Region 1.6 | 3,120,929 | 3,162,155 | 41.23 | arylpolyene | APE Ec | 10 |
|  | Region 2.1 | 122,545 | 174,560 | 52.02 | NRPS | acyldepsipeptide 1 | 10 |
|  | Region 2.2 | 373,179 | 418,064 | 44.89 | arylpolyene | APE Vf | 40 |
|  | Region 2.3 | 480,366 | 497,082 | 16.72 | siderophore | staphylobactin | 18 |
|  | Region 2.4 | 641,473 | 720,018 | 78.55 | NRPS,T1PKS | ralsolamycin | 100 |
|  | Region 2.5 | 858,530 | 879,084 | 20.55 | hserlactone | obafluorin | 14 |
|  | Region 2.6 | 1,251,111 | 1,261,989 | 10.88 | RiPP-like |  |  |
|  | Region 2.7 | 1,580,515 | 1,621,615 | 41.10 | T3PKS |  |  |
|  | Region 2.8 | 1,791,518 | 1,811,838 | 20.32 | RRE-containing |  |  |
| *R. pseudosolanacearum* RUN2279 | Region 1.1 | 156,281 | 176,877 | 20.60 | hserlactone |  |  |
|  | Region 1.2 | 1,950,340 | 1,971,212 | 20.87 | terpene |  |  |
|  | Region 1.3 | 2,074,592 | 2,146,231 | 71.64 | T1PKS,NRPS | micacocidin | 100 |
|  | Region 1.4 | 3,076,196 | 3,122,663 | 46.47 | NRPS |  |  |
|  | Region 1.5 | 3,372,934 | 3,414,160 | 41.23 | arylpolyene | APE Vf | 10 |
|  | Region 2.1 | 375,023 | 443,709 | 68.69 | NRPS,T1PKS,NRPS-like | ralsolamycin | 100 |
|  | Region 2.2 | 575,523 | 592,239 | 16.72 | siderophore | staphylobactin | 18 |
|  | Region 2.3 | 623,774 | 668,659 | 44.89 | arylpolyene | APE Vf | 35 |
|  | Region 2.4 | 831,831 | 917,016 | 85.19 | T1PKS,NRPS | crocagin A / crocagin B | 36 |
|  | Region 2.5 | 1,251,716 | 1,272,036 | 20.32 | RRE-containing |  |  |
|  | Region 2.6 | 1,810,609 | 1,821,487 | 10.88 | RiPP-like |  |  |
| *R. pseudosolanacearum* RUN2474 | Region 1.1 | 161,747 | 182,361 | 20.61 | hserlactone |  |  |
|  | Region 1.2 | 1,829,549 | 1,850,421 | 20.87 | terpene |  |  |
|  | Region 1.3 | 2,004,738 | 2,076,353 | 71.62 | T1PKS,NRPS | micacocidin | 100 |
|  | Region 1.4 | 2,950,045 | 2,996,512 | 46.47 | NRPS |  |  |
|  | Region 1.5 | 3,249,169 | 3,290,395 | 41.23 | arylpolyene | APE Vf | 10 |
|  | Region 2.1 | 318,568 | 403,189 | 84.62 | NRPS,T1PKS | ralsolamycin | 100 |
|  | Region 2.2 | 537,704 | 554,420 | 16.72 | siderophore | staphylobactin | 18 |
|  | Region 2.3 | 584,874 | 629,759 | 44.89 | arylpolyene | APE Vf | 40 |
|  | Region 2.4 | 805,659 | 893,639 | 87.98 | T1PKS,NRPS | crocagin A / crocagin B | 36 |
|  | Region 2.5 | 1,227,875 | 1,248,195 | 20.32 | RRE-containing |  |  |
|  | Region 2.6 | 1,838,002 | 1,848,880 | 10.88 | RiPP-like |  |  |
| *R. pseudosolanacearum* UW386 | Region 1.1 | 1,882,152 | 1,923,258 | 41.11 | T3PKS | chejuenolide A / chejuenolide B | 7 |
|  | Region 1.2 | 2,020,586 | 2,042,829 | 22.24 | LAP |  |  |
|  | Region 1.3 | 2,127,159 | 2,148,031 | 20.87 | terpene |  |  |
|  | Region 1.4 | 2,242,551 | 2,315,323 | 72.77 | NRPS,T1PKS | micacocidin | 100 |
|  | Region 1.5 | 2,873,822 | 2,894,436 | 20.61 | hserlactone |  |  |
|  | Region 1.6 | 3,450,992 | 3,492,227 | 41.24 | arylpolyene | APE Vf | 10 |
|  | Region 2.1 | 1 | 109,849 | 109.85 | transAT-PKS,NRPS-like,T1PKS | rhizoxin A | 100 |
|  | Region 2.2 | 438,294 | 455,010 | 16.72 | siderophore | staphylobactin | 18 |
|  | Region 2.3 | 483,094 | 527,979 | 44.89 | arylpolyene | APE Vf | 35 |
|  | Region 2.4 | 732,041 | 783,600 | 51.56 | NRPS | marformycin A / marformycin B / marformycin C / marformycin D / marformycin E / marformycin F | 8 |
|  | Region 2.5 | 1,137,627 | 1,179,550 | 41.92 | NRPS-like |  |  |
|  | Region 2.6 | 1,637,686 | 1,648,564 | 10.88 | RiPP-like |  |  |
|  | Region 2.7 | 1,758,423 | 1,830,097 | 71.67 | transAT-PKS,NRPS-like,T1PKS,NRPS |  |  |
| *R. solanacearum* K60-1^T^ | Region 1.1 | 1,291,962 | 1,333,188 | 41.23 | arylpolyene |  |  |
|  | Region 1.2 | 2,007,190 | 2,018,197 | 11.01 | RiPP-like |  |  |
|  | Region 1.3 | 2,025,361 | 2,045,975 | 20.61 | hserlactone |  |  |
|  | Region 1.4 | 3,767,518 | 3,788,390 | 20.87 | terpene |  |  |
|  | Region 2.1 | 154,489 | 205,642 | 51.15 | NRPS | marformycin A / marformycin B / marformycin C / marformycin D / marformycin E / marformycin F | 8 |
|  | Region 2.2 | 373,437 | 418,340 | 44.90 | arylpolyene | aryl polyenes | 44 |
|  | Region 2.3 | 449,925 | 493,417 | 43.49 | siderophore,NRPS-like | staphylobactin | 18 |
|  | Region 2.4 | 941,994 | 1,061,582 | 119.59 | NRPS-like,transAT-PKS | rhizoxin A | 100 |
|  | Region 2.5 | 1,156,299 | 1,213,488 | 57.19 | NRPS,T1PKS | yersiniabactin | 11 |
|  | Region 2.6 | 1,236,085 | 1,246,963 | 10.88 | RiPP-like |  |  |
| *R. solanacearum* CFBP2957 | Region 1.1 | 176,439 | 187,449 | 11.01 | RiPP-like |  |  |
|  | Region 1.2 | 194,630 | 215,244 | 20.61 | hserlactone |  |  |
|  | Region 1.3 | 1,705,344 | 1,726,216 | 20.87 | terpene |  |  |
|  | Region 1.4 | 2,986,830 | 3,028,068 | 41.24 | arylpolyene |  |  |
|  | Region 2.1 | 141,351 | 195,820 | 54.47 | NRPS | marformycin A / marformycin B / marformycin C / marformycin D / marformycin E / marformycin F | 8 |
|  | Region 2.2 | 405,111 | 450,014 | 44.90 | arylpolyene | aryl polyenes | 44 |
|  | Region 2.3 | 481,606 | 498,346 | 16.74 | siderophore | staphylobactin | 18 |
|  | Region 2.4 | 1,056,754 | 1,176,576 | 119.82 | NRPS-like,transAT-PKS | rhizoxin A | 100 |
|  | Region 2.5 | 1,290,320 | 1,347,500 | 57.18 | NRPS,T1PKS | yersiniabactin | 11 |
|  | Region 2.6 | 1,375,959 | 1,386,837 | 10.88 | RiPP-like |  |  |
| *R. solanacearum* Po82 | Region 1.1 | 163,030 | 174,040 | 11.01 | RiPP-like |  |  |
|  | Region 1.2 | 181,210 | 201,824 | 20.61 | hserlactone |  |  |
|  | Region 1.3 | 1,797,786 | 1,818,658 | 20.87 | terpene |  |  |
|  | Region 1.4 | 3,081,235 | 3,122,566 | 41.33 | arylpolyene |  |  |
|  | Region 2.1 | 156,152 | 203,691 | 47.54 | NRPS | cyclomarin D | 8 |
|  | Region 2.2 | 1,042,095 | 1,082,377 | 40.28 | NRPS-like | JBIR-06 | 16 |
|  | Region 2.3 | 1,350,156 | 1,426,812 | 76.66 | transAT-PKS | rhizoxin A | 66 |
|  | Region 2.4 | 1,493,084 | 1,550,273 | 57.19 | NRPS,T1PKS | yersiniabactin | 11 |
|  | Region 2.5 | 1,574,612 | 1,585,490 | 10.88 | RiPP-like |  |  |
|  | Region 2.6 | 1,643,015 | 1,697,467 | 54.45 | siderophore,NRPS-like | staphylobactin | 18 |
| *R. syzygii* LLRS-1 | Region 1.1 | 144,175 | 164,789 | 20.61 | hserlactone |  |  |
|  | Region 1.2 | 1,726,029 | 1,746,901 | 20.87 | terpene |  |  |
|  | Region 1.3 | 1,882,698 | 1,955,406 | 72.71 | NRPS,T1PKS | micacocidin | 100 |
|  | Region 1.4 | 3,215,261 | 3,256,493 | 41.23 | arylpolyene | xanthomonadin I | 14 |
|  | Region 2.1 | 395,881 | 416,435 | 20.55 | hserlactone | obafluorin | 14 |
|  | Region 2.2 | 716,294 | 844,984 | 128.69 | transAT-PKS,NRPS-like,siderophore | rhizoxin A | 100 |
|  | Region 2.3 | 1,089,654 | 1,145,004 | 55.35 | NRPS | marformycin A / marformycin B / marformycin C / marformycin D / marformycin E / marformycin F | 12 |
|  | Region 2.4 | 2,025,689 | 2,036,567 | 10.88 | RiPP-like |  |  |
| *R. syzygii* PSI 7^T^ | Region 1.1 | 163,427 | 184,041 | 20.61 | hserlactone |  |  |
|  | Region 1.2 | 1,756,064 | 1,776,897 | 20.83 | terpene |  |  |
|  | Region 1.3 | 1,889,757 | 1,962,466 | 72.71 | NRPS,T1PKS | micacocidin | 100 |
|  | Region 1.4 | 3,059,946 | 3,101,178 | 41.23 | arylpolyene | xanthomonadin I | 14 |
|  | Region 2.1 | 107,696 | 156,674 | 48.98 | NRPS | rhizomide A / rhizomide B / rhizomide C | 100 |
|  | Region 2.2 | 435,759 | 565,264 | 129.51 | siderophore,NRPS-like,transAT-PKS | rhizoxin A | 100 |
|  | Region 2.3 | 853,263 | 873,817 | 20.55 | hserlactone | obafluorin | 14 |
|  | Region 2.4 | 1,286,617 | 1,297,495 | 10.88 | RiPP-like |  |  |
|  | Region 2.5 | 1,863,323 | 1,884,192 | 20.87 | furan | O-antigen | 10 |
|  | Region 2.6 | 1,890,343 | 1,910,663 | 20.32 | RRE-containing |  |  |
| *R. syzygii* subsp. *celebesensis* UGMSS_Db01 | Region 1.1 | 167,276 | 208,508 | 41.23 | arylpolyene | xanthomonadin I | 14 |
|  | Region 1.2 | 264,602 | 326,299 | 61.70 | NRPS,hserlactone | marformycin A / marformycin B / marformycin C / marformycin D / marformycin E / marformycin F | 8 |
|  | Region 1.3 | 1,343,264 | 1,364,097 | 20.83 | terpene |  |  |
|  | Region 1.4 | 2,466,506 | 2,538,472 | 71.97 | NRPS,T1PKS,NRPS-like | micacocidin | 100 |
|  | Region 1.5 | 3,004,974 | 3,135,336 | 130.36 | transAT-PKS,NRPS-like,siderophore | rhizoxin A | 100 |
|  | Region 2.1 | 1,173,127 | 1,184,005 | 10.88 | RiPP-like |  |  |
| *R. chuxiongensis* 21YRMH01-3^T^ | Region 1.1 | 276,443 | 287,312 | 10.87 | RiPP-like |  |  |
|  | Region 2.1 | 500,264 | 525,290 | 25.03 | betalactone |  |  |
|  | Region 2.2 | 568,720 | 580,687 | 11.97 | siderophore |  |  |
|  | Region 2.3 | 665,913 | 707,130 | 41.22 | arylpolyene |  |  |
|  | Region 4.1 | 290,001 | 312,138 | 22.14 | redox-cofactor |  |  |
|  | Region 5.1 | 176,705 | 197,538 | 20.83 | terpene |  |  |
|  | Region 7.1 | 20,782 | 46,282 | 25.50 | betalactone |  |  |
| *R. insidiosa* CCUG 46789^T^ | Region 1.1 | 745,129 | 770,141 | 25.01 | betalactone |  |  |
|  | Region 1.2 | 934,302 | 975,534 | 41.23 | arylpolyene |  |  |
|  | Region 1.3 | 2,040,564 | 2,061,397 | 20.83 | terpene |  |  |
|  | Region 1.4 | 2,072,745 | 2,084,631 | 11.89 | siderophore | putrebactin / avaroferrin | 30 |
|  | Region 3.1 | 291,430 | 313,549 | 22.12 | redox-cofactor | lankacidin C | 13 |
|  | Region 5.1 | 104,304 | 115,170 | 10.87 | RiPP-like |  |  |
| *R. mannitolilytica* LMG 6866^T^ | Region 1.1 | 879,438 | 924,308 | 44.87 | arylpolyene | APE Vf | 35 |
|  | Region 13.1 | 87,427 | 98,296 | 10.87 | RiPP-like |  |  |
|  | Region 3.1 | 69,421 | 90,293 | 20.87 | terpene |  |  |
|  | Region 3.2 | 94,254 | 106,134 | 11.88 | siderophore | putrebactin / avaroferrin | 30 |
|  | Region 4.1 | 65,843 | 87,980 | 22.14 | redox-cofactor | lankacidin C | 13 |
|  | Region 5.1 | 191,115 | 232,371 | 41.26 | arylpolyene | APE Vf | 10 |
| *R. mojiangensis* 21MJYT02-10^T^ | Region 1.1 | 539,958 | 562,095 | 22.14 | redox-cofactor |  |  |
|  | Region 1.2 | 1,243,264 | 1,264,097 | 20.83 | terpene |  |  |
|  | Region 1.3 | 1,272,752 | 1,285,259 | 12.51 | siderophore | putrebactin / avaroferrin | 30 |
|  | Region 2.1 | 206,559 | 247,776 | 41.22 | arylpolyene | APE Vf | 10 |
|  | Region 2.2 | 341,179 | 353,155 | 11.98 | siderophore |  |  |
|  | Region 2.3 | 395,871 | 420,898 | 25.03 | betalactone |  |  |
|  | Region 4.1 | 425,153 | 470,017 | 44.86 | arylpolyene | APE Vf | 35 |
|  | Region 8.1 | 6,589 | 17,458 | 10.87 | RiPP-like |  |  |
| *R. pickettii* K-288^T^ | Region 1.1 | 125,693 | 150,720 | 25.03 | betalactone |  |  |
|  | Region 1.2 | 193,774 | 205,741 | 11.97 | siderophore |  |  |
|  | Region 1.3 | 294,479 | 335,696 | 41.22 | arylpolyene | APE Vf | 10 |
|  | Region 1.4 | 1,586,897 | 1,607,730 | 20.83 | terpene |  |  |
|  | Region 1.5 | 1,692,520 | 1,740,190 | 47.67 | T1PKS | lipopolysaccharide | 8 |
|  | Region 2.1 | 1,065,544 | 1,076,413 | 10.87 | RiPP-like |  |  |
| *R. soli* 21MJYT02-11^T^ | Region 1.1 | 641,625 | 682,863 | 41.24 | arylpolyene |  |  |
|  | Region 10.1 | 1 | 14,923 | 14.92 | RRE-containing | lankacidin C | 13 |
|  | Region 14.1 | 6,363 | 17,232 | 10.87 | RiPP-like |  |  |
|  | Region 4.1 | 106,156 | 126,998 | 20.84 | terpene |  |  |
|  | Region 7.1 | 79,123 | 124,047 | 44.92 | arylpolyene | APE Vf | 40 |
| *R. wenshanensis* 56D2^T^ | Region 1.1 | 263,775 | 274,644 | 10.87 | RiPP-like |  |  |
|  | Region 2.1 | 121,742 | 146,769 | 25.03 | betalactone |  |  |
|  | Region 2.2 | 190,196 | 202,163 | 11.97 | siderophore |  |  |
|  | Region 2.3 | 332,980 | 374,197 | 41.22 | arylpolyene | APE Vf | 10 |
|  | Region 2.4 | 1,650,405 | 1,671,238 | 20.83 | terpene |  |  |
|  | Region 2.5 | 2,273,559 | 2,295,696 | 22.14 | redox-cofactor |  |  |

Note: betalactone, beta-lactone-containing protease inhibitor; hserlactone, homoserine lactone; NRPS, non-ribosomal peptide synthetase (NRPS); RiPP-like, ribosomally synthesized and post-translationally modified peptide product-like; T1PKS, type I polyketide synthase.

Supplementary Table 5 Table traits characterizing strains obtained in this study and their closely related type strains of the genus *Ralstonia*

Strains: 1. RS^T^; 2. GMI1000; 3. CQPS-1; 4. *R. pseudosolanacearum* LMG 9673^T^; 5. *R. solanacearum* LMG 2299^T^; 6. *R. syzygii* subsp. *syzygii* LMG 10661^T^; 7. *R. syzygii* subsp. *celebesensis* LMG 27706^T^; 8. *R. syzygii* subsp. *indonesiensis* LMG 27703^T^. Data of strains marked with ^*^ were derived from Lu et al. (2021). Symbols: +, positive; -, negative; -/+, weak activity.

| Characteristics | 1 | 2 | 3 | 4 | 5 | 6 | 7 | 8 |
| --- | --- | --- | --- | --- | --- | --- | --- | --- |
| API 20 NE: |  |  |  |  |  |  |  |  |
| Potassium nitrate | + | + | + | + | + | - | - | + |
| D-Glucose | - | - | - | + | + | - | - | + |
| D-Glucose | + | + | + | + | + | - | - | + |
| D-Mannitol | +/- | - | - | - | - | - | - | - |
| N-Acetyl- glucosamine | - | - | -/+ | - | - | - | - | - |
| Potassium gluconate | +/- | + | + | +/- | + | - | - | + |
| Capric acid | + | - | + | - | - | - | - | + |
| Malic acid | + | + | + | + | + | - | - | + |
| Trisodium citrate | + | + | +/- | + | + | - | - | + |
| Phenylacetic acid | - | - | +/- | - | - | - | - | - |
| API ZYM: |  |  |  |  |  |  |  |  |
| Alkaline phosphatase | - | + | - | - | + | + | + | - |
| Esterase(C4) | + | - | + | + | + | + | + | + |
| Lipase(C14) | - | - | - | + | - | - | - | - |
| Leucine arylamidase | + | + | + | - | + | + | - | + |
| Acid phosphatase | + | + | + | + | - | - | + | + |
| Naphthol-AS-BI-phosphohydrolase | - | - | - | - | + | - | + | + |
| β-galactosidase | - | - | + | - | - | - | - | - |
| α-glucosidase | - | - | - | - | - | - | + | - |
| Gen III: |  |  |  |  |  |  |  |  |
| Dextrin | ﹣ | ﹣ | ﹣ | ﹣/+ | ﹣/+ | ﹣ | ﹣ | ﹣ |
| pH 6 | +/- | ﹣ | + | +/﹣ | +/﹣ | ﹣ | ﹣ | ﹣ |
| D-Melibiose | ﹣ | ﹣ | ﹣ | ﹣ | ﹣ | ﹣ | ﹣ | ﹣/+ |
| α-D-Glucose | ﹣ | ﹣ | ﹣ | ﹣/+ | +/﹣ | ﹣ | ﹣ | ﹣/+ |
| D-Mannose | ﹣ | ﹣ | ﹣ | ﹣/+ | ﹣ | ﹣ | ﹣ | ﹣/+ |
| D-Fructose | ﹣/+ | ﹣ | ﹣ | +/﹣ | +/﹣ | ﹣ | ﹣ | ﹣/+ |
| D-Galactose | ﹣/+ | ﹣ | ﹣ | ﹣/+ | ﹣/+ | ﹣ | ﹣ | ﹣/+ |
| 3-Methyl Glucose | ﹣ | ﹣ | ﹣ | ﹣/+ | ﹣/+ | ﹣ | ﹣/+ | ﹣/+ |
| D-Fucose | ﹣/+ | ﹣ | ﹣ | ﹣/+ | ﹣/+ | ﹣ | ﹣/+ | ﹣/+ |
| L-Fucose | ﹣ | ﹣ | ﹣ | ﹣/+ | ﹣ | ﹣ | ﹣/+ | ﹣/+ |
| L-Rhamnose | ﹣ | ﹣ | ﹣ | ﹣ | ﹣ | ﹣ | ﹣/+ | ﹣ |
| D-Glucose- 6-PO4 | ﹣/+ | ﹣ | ﹣ | ﹣ | ﹣/+ | ﹣ | ﹣ | ﹣ |
| D-Fructose- 6-PO4 | ﹣/+ | ﹣/+ | ﹣/+ | ﹣/+ | ﹣/+ | ﹣ | ﹣/+ | ﹣ |
| D-Aspartic Acid | ﹣ | ﹣ | ﹣ | ﹣ | ﹣/+ | ﹣ | ﹣ | ﹣ |
| Rifamycin SV | + | +/- | + | + | + | ﹣ | ﹣ | +/﹣ |
| L-Aspartic Acid | ﹣ | ﹣ | ﹣ | +/﹣ | +/﹣ | ﹣ | ﹣ | ﹣ |
| L-Glutamic Acid | ﹣ | ﹣ | ﹣ | +/﹣ | +/﹣ | ﹣ | ﹣ | ﹣ |
| L-Histidine | ﹣ | ﹣ | ﹣ | ﹣ | ﹣/+ | ﹣ | ﹣ | ﹣/+ |
| Lincomycin | + | +/- | + | + | + | ﹣ | ﹣ | +/﹣ |
| Niaproof 4 | ﹣ | ﹣ | ﹣ | ﹣ | ﹣ | ﹣ | +/- | ﹣ |
| Pectin | ﹣ | ﹣ | ﹣ | + | +/﹣ | ﹣ | ﹣ | ﹣/+ |
| D-Galacturonic Acid | +/- | +/- | +/- | + | +/﹣ | ﹣ | + | ﹣/+ |
| L-Galactonic Acid Lactone | +/- | +/- | +/- | +/﹣ | +/﹣ | ﹣ | ﹣ | ﹣/+ |
| D-Glucuronic Acid | + | +/- | +/- | + | +/﹣ | ﹣ | ﹣/+ | ﹣/+ |
| Vancomycin | + | +/- | + | + | +/﹣ | ﹣ | ﹣ | +/﹣ |
| Tetrazolium Violet | + | + | + | + | + | ﹣ | + | + |
| Tetrazolium Blue | + | +/- | + | + | + | ﹣ | ﹣ | + |
| Citric Acid | ﹣ | ﹣ | ﹣ | ﹣/+ | +/﹣ | ﹣ | ﹣ | ﹣ |
| α-Keto-Glutaric Acid | ﹣ | ﹣ | ﹣ | ﹣ | +/﹣ | ﹣ | ﹣ | ﹣ |
| L-Malic Acid | ﹣ | ﹣ | ﹣ | ﹣/+ | +/﹣ | ﹣ | ﹣ | ﹣ |
| Nalidixic Acid | ﹣ | ﹣ | ﹣ | ﹣/+ | ﹣ | ﹣ | ﹣ | ﹣ |
| Potassium Tellurite | + | ﹣/+ | + | + | +/﹣ | ﹣ | ﹣ | ﹣/+ |
| Tween 40 | ﹣ | ﹣ | ﹣ | ﹣/+ | ﹣/+ | ﹣ | ﹣ | ﹣ |
| γ-Amino-Butryric Acid | ﹣ | ﹣ | ﹣ | ﹣ | ﹣/+ | ﹣ | ﹣ | ﹣ |
| β-Hydroxy-D,L- Butyric Acid | ﹣ | ﹣ | ﹣ | ﹣ | +/﹣ | ﹣ | ﹣ | ﹣ |
| Acetoacetic Acid | ﹣ | ﹣ | ﹣ | ﹣ | +/﹣ | ﹣ | ﹣ | ﹣ |
| Propionic Acid | ﹣ | ﹣ | ﹣ | ﹣ | ﹣ | ﹣ | ﹣ | ﹣ |
| Acetic Acid | ﹣ | ﹣ | ﹣ | ﹣/+ | +/﹣ | ﹣ | ﹣ | ﹣/+ |
| Formic Acid | ﹣ | ﹣ | ﹣ | ﹣ | ﹣ | ﹣ | ﹣ | ﹣ |
| Aztreonam | ﹣/+ | ﹣ | ﹣ | +/﹣ | +/﹣ | ﹣ | ﹣ | ﹣ |

**REFERENCES**

Blin, K., Shaw, S., Kloosterman, A. M., Charlop-Powers, Z., van Wezel, G. P., Medema, Marnix H., et al. (2021). antiSMASH 6.0: Improving cluster detection and comparison capabilities. *Nucleic Acids Research* 49, W29-W35. doi: 10.1093/nar/gkab335.

Fluit, A. C., Bayjanov, J. R., Aguilar, M. D., Cantón, R., Tunney, M. M., Elborn, J. S., et al. (2021). Characterization of clinical *Ralstonia* strains and their taxonomic position. *Antonie van Leeuwenhoek* 114, 1721-1733. doi: 10.1007/s10482-021-01637-0.

Jain, C., Rodriguez-R, L. M., Phillippy, A. M., Konstantinidis, K. T., and Aluru, S. (2018). High throughput ANI analysis of 90K prokaryotic genomes reveals clear species boundaries. *Nature Communications* 9, 5114. doi: 10.1038/s41467-018-07641-9.

Meier-Kolthoff, J. P., Carbasse, J. S., Peinado-Olarte, R. L., and Goker, M. (2022). TYGS and LPSN: A database tandem for fast and reliable genome-based classification and nomenclature of prokaryotes. *Nucleic Acids Research* 50, D801-D807. doi: 10.1093/nar/gkab902.

Meier-Kolthoff, J. P., and Göker, M. (2019). TYGS is an automated high-throughput platform for state-of-the-art genome-based taxonomy. *Nature Communications* 10, 2182. doi: 10.1038/s41467-019-10210-3.

Parks, D. H., Chuvochina, M., Rinke, C., Mussig, A. J., Chaumeil, P.-A., and Hugenholtz, P. (2021). GTDB: An ongoing census of bacterial and archaeal diversity through a phylogenetically consistent, rank normalized and complete genome-based taxonomy. *Nucleic Acids Research* 50, D785-D794. doi: 10.1093/nar/gkab776.

Richter, M., Rosselló-Móra, R., Oliver Glöckner, F., and Peplies, J. (2015). JSpeciesWS: A web server for prokaryotic species circumscription based on pairwise genome comparison. *Bioinformatics* 32, 929-931. doi: 10.1093/bioinformatics/btv681.
